# Resistance Gene-Guided Genome Mining Reveals RPL10 as the Target of Ligustrone A

**DOI:** 10.1021/jacs.5c03802

**Published:** 2025-06-16

**Authors:** Danielle A. Yee, Ross D. Overacker, Michelle F. Grau, Amber Cornelius, Adrianne Pigula, Daniel Mummau, Thomas Pavey, Cynthia Bailey, Clarence Hue Lok Yeung, Lawrence S. Hon, Joseph E. Spraker, Sheena Li, Bruno Perlatti, Samuel K. Oteng-Pabi, Henry H. Le, Colin J. B. Harvey

**Affiliations:** Hexagon Bio, Menlo Park, California 94025, United States

## Abstract

The ribosome is essential for protein synthesis and targeting its function serves as a platform for many therapies. Resistance gene-guided genome mining for *a priori* target identification has emerged as a useful workflow in natural product drug discovery, allowing for identification of a compound’s target before the time-consuming work of isolation and characterization is performed. In this work, we identify the previously unknown *lig* biosynthetic gene cluster (BGC) which contains a predicted resistance gene that shares sequence identity with the gene for human ribosomal protein RPL10. Through both heterologous expression and *in situ* BGC engineering, we identify ligustrone A (**1**) as this BGC’s product. Utilizing chemical genetics and chemoproteomics, we validate that **1** engages RPL10. Taken together, via resistance gene-guided mining we both discover a new target for a known natural product and elucidate its biosynthesis.

## Graphical abstract

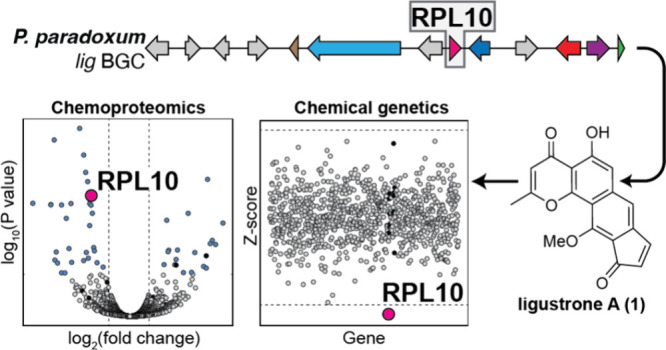

Inhibition of ribosomal activity is a promising approach for developing anticancer, antifungal, and antibiotic compounds.^1–4^ Ribosomal proteins, numbering over 50, serve diverse functions, including structural scaffolding and precise positioning of catalytic domains to ensure high-fidelity protein translation.^5^ As most ribosomal proteins are essential for activity, inhibiting them has been a major focus of therapeutic research.

Natural products, often discovered through traditional screening, dominate the repertoire of protein translation inhibitors. However, this screening is resource intensive, as natural product isolation and characterization typically precede target identification. Resistance gene-guided discovery offers an alternative by mining biosynthetic gene clusters (BGCs) to identify those that encode genes that are homologous to targets-of-interest thereby linking the products of BGCs with their potential target. This approach prioritizes target determination before metabolite isolation.^6–8^

In fungi, this method was first appreciated with a 3-hydroxy-3-methylglutaryl coenzyme A (HMG-CoA) reductase gene within the lovastatin BGC of Aspergillus terreus. Subsequent discoveries have linked BGCs to inhibitors of ergosterol and sphingolipid biosynthesis, glycolysis, oxidative phosphorylation, the proteasome, calcineurin, and cyclin-dependent kinases.^9–16^

Owing to the therapeutic value of novel protein translation inhibitors, we mined our database of fungal BGCs for genes homologous to those of ribosomal proteins. This analysis yielded a family of BGCs, present in both Penicillium paradoxum (*lig* BGC) and Botryosphaeria dothidea (*bdlig* BGC) (Figure 1A) containing a core nonreducing polyketide synthase (NRPKS, Figure S1A) gene and a homolog of *RPL10* (also recognized as *ul16*), the gene for large-subunit ribosomal protein RPL10, which, when overexpressed, is reported to play a role in the proliferation of ovarian and pancreatic cancers.^17,18^ Canonically, ribosomal protein genes are not present in BGCs, making this a strong candidate for a resistance gene.

**Figure 1:**
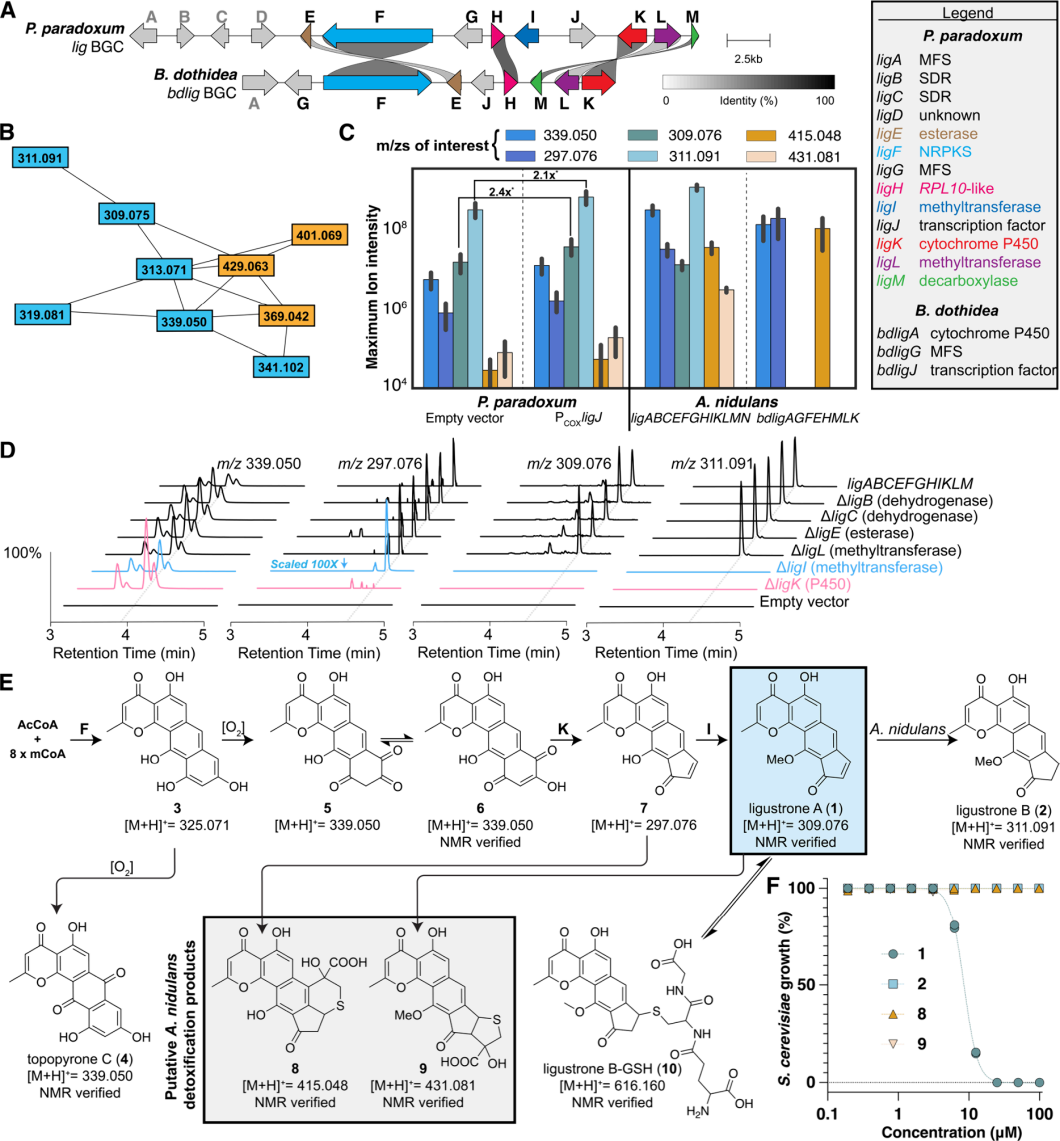
Biosynthesis of 1 and 2. (A) *lig* and *bdlig* BGCs in the genomes of P. paradoxum and B. dothidea. Links between homologous genes (≥20% amino acid identity) are depicted.^33^ (B) Molecular network of BGC related features both with (orange) and without (blue) sulfur. (C) Intensities of select BGC related m/zs across producing strains reported as mean ± SD (*n* = 6 clones per strain, *P value <0.01). (D) High-resolution mass spectrometry extracted ion chromatogram (EIC) traces of key BGC derived signals in cultures of A. nidulans expressing single gene knockout versions of the *lig* BGC. (E) Proposed biosynthesis of the ligustrones. (F) Inhibition of S. cerevisiae growth by products of the *lig* BGC. Biological replicates shown for all points (*n* = 2).

To elucidate the products of this BGC family, we employed both heterologous expression and *in situ* activation via transcription factor overexpression.^19^ In the heterologous expression approach, the *lig* and *bdlig* BGCs were fully refactored and transformed into Aspergillus nidulans.^20^ Fermentation and untargeted metabolomic analysis of strains expressing the *lig* BGC identified 15 significant metabolic features (Figure S1B) that were absent in untransformed A. nidulans, suggesting the BGC was functionally expressed in the heterologous host.^21^ Among these features, five exhibited isotopic spectra consistent with sulfur-containing metabolites.^22^ These features were linked to other nonsulfur-containing features through molecular networking analysis using MS2Deepscore (Figure 1B, threshold score = 0.8).^23^ Several features identified in heterologous strains expressing the *lig* BGC were also observed in strains expressing the refactored *bdlig* BGC (Figure 1C). Additionally, these features were produced at significantly higher levels in P. paradoxum strains transformed with a plasmid encoding *ligJ*—the gene for a BGC-specific transcription factor driven by a strong constitutive promoter (P_COX_)—compared to wild-type P. paradoxum (Figure 1C). Transcriptomic analysis of P. paradoxum strains containing plasmids expressing *ligJ* both confirmed cluster activation and provided support for *ligA* to *ligM* being the complete BGC (Figure S2). Collectively, these findings provide strong evidence that the identified features are products of the *lig* BGC.

Large scale fermentation of A. nidulans strains expressing the *lig* BGC enabled isolation of six metabolites (three with sulfur and three without) in quantities sufficient for NMR characterization. The three metabolites without sulfur were the previously reported ligustrone B (**2**, Figure 1D, Table S3, Figures S9, S10), topopyrone C (**4**, Figure 1D, Table S4, Figures S11, S12), and **6**, (Figure 1D, Table S5, Figures S13–S16). The three metabolites containing sulfur were variants of **2** with sulfur attached to carbon-8 (**8**-**10**). In each of **8** (Figure 1D, Table S6, Figures S17–S21) and **9** (Figure 1D, Table S7, Figures S22–S26), the origin of the sulfur is likely addition of a cysteine-derived metabolite, such as mercaptopyruvate followed by cyclization while **10** (Table S8, Figures S27–S31) is an adduct of glutathione.^24^ Intriguingly, the origin of the metabolites containing sulfur is likely **1** (Table S2, Figures S7, S8) followed by thia-Michael addition; a hypothesis supported by near complete conversion of **1** to **10** upon incubation with an excess of glutathione in PBS buffer for 1 h **(** Figure S3). Thia-Michael additions are a known detoxification pathway in Aspergillus species, suggesting that **1** was likely produced in higher quantities, but is quickly converted to these presumably inert adducts.^24,25^ Indeed, while **1** demonstrates significant antifungal activity against *S. cerevisiae* (IC_50_ = 9 μM), none of **8**, **9**, and **2** show inhibition of fungal growth at concentrations up to 100 μM (Figure 1F). That these thiol adducts are a result of detoxification by the heterologous host is further supported by their presence only at very low levels in cultures of P. paradoxum, suggesting the native fungus is better able to tolerate the toxin (Figure 1C). To our knowledge, this is the first identification of the ligustrone BGC.

Given the observed thiol-reactivity of **1**, it is notable that RPL10 contains a reactive cysteine residue (C105) that is highly conserved across species, including P. paradoxum and B. dothidea.^26^ However, despite very strong conservation in the structures as predicted by AlphaFold (LigH vs S. cerevisiae RPL10, RMSD = 0.31 Å, Figure S2B),^27^ comparison of the products of putative resistance genes *ligH* and *bdligH* with canonical RPL10 proteins reveals mutations at this key residue in both (C105V in LigJ, C105M in bdLigJ, Figure S5A).

Using this heterologous expression system, we sought to better characterize the biosynthesis of cluster-derived metabolites. Biosynthesis of **1** is likely initiated by LigF, which uses acetyl-Coa (AcCoA) and malonyl-CoA (mCoA) to produce **3**, an anthracene similar to the topopyrones. Indeed, LigF and the topopyrone NRPKS (PKN2) share 58% amino acid identity.^26^ As noted previously, we identified topopyrone C (**4**) as a product of the BGC, likely as the result of the nonenzymatic oxidation of **3**.^28^ Following the release of **3** from LigF, we envision oxidation to produce a 1,2-dione intermediate **5**, the tautomer of which, **6**, was isolated and characterized. This oxidation is not catalyzed by LigK, as **6** persists in *ΔligK* strains (Figure 1D). Next, we propose conversion of **5** to **7** through a ring contraction, mediated by the cytochrome P450 LigK as removal of *ligK* was sufficient to prevent conversion of **5** to **7** (Figure 1D). While a similar conversion is catalyzed by CapL, a flavin dependent monooxygenase (FMO) in AS2077715 biosynthesis, LigK is unrelated to CapL, demonstrating a unique means of oxidative ring contraction in ligustrone biosynthesis.^29^

To identify the methyltransferase responsible for the conversion of **7** to **1**, we individually deleted the methyltransferase genes *ligI* and *ligL*. Deletion of *ligL* had no effect on the production of **1** or **2**, while deletion of *ligI* abolished the production of both **1** and **2**, resulting in the significant accumulation of the unmethylated intermediate **7**. These findings confirm that LigI catalyzes O-methylation of **7** to produce **1** (Figure 1D). Notably, the *bdlig* BGC lacks a homolog of *ligI*, and consistent with the role of LigI in O-methylation, neither **1** nor **2** were detected in cultures of A. nidulans expressing the *bdlig* BGC (*m*/*z* = 309.076, 311.091, Figure 1C) while **7** accumulated at levels exceeding those observed in strains expressing the *lig* BGC (*m*/*z* = 297.076, Figure 1C).

While *ligL* deletion did not noticeably perturb the metabolite profile, LigL bears modest similarity to CapK (30% amino acid identity), a methyltransferase from the AS2077715 BGC that, while not essential for AS2077715 biosynthesis, does help to both accelerate and direct the stereochemistry of the ring contraction reaction observed in that pathway, leading us to speculate that perhaps LigL plays a similar role in the conversion of **5** to **7**.^29^

Finally, we noted extensive reduction of **1** to **2**, a transformation potentially catalyzed by LigB or LigC, the products of two short-chain dehydrogenase/reductase (SDR) genes within the *lig* BGC. Individual deletions of both SDR genes from our heterologous expression system had no effect on the metabolite profile (Figure 1D) while incubation of **1** in a culture of our untransformed heterologous host led to significant conversion of **1** to **2**, demonstrating that this reduction can be catalyzed by enzymes endogenous to A. nidulans (Figure S4).

With **1** established as the product of the *lig* BGC, we first sought to determine whether it interacts with RPL10 by utilizing a well-characterized yeast chemical genetic screen in which a defined pool of 914 barcoded S. cerevisiae heterozygous diploid deletion mutants with broad coverage of the essential genome are subjected to either vehicle or compound.^30^ The barcodes in both pools are sequenced to determine which mutants are over- and under-represented in the compound treated population. Those mutants whose barcodes are enriched in the treated pool are resistant to treatment while those that are depleted are sensitive, suggesting haploinsufficiency. Upon treatment of this mutant pool with 250 μM **1**, RPL10 emerges as the sole significant sensitized chemical genetic interaction (Figure 2A), a result strongly suggestive of RPL10 engagement. The chemical genetic interaction score of **1** with RPL10 shows a strong dose dependence and becomes significant at concentrations demonstrating significant growth inhibition of the pool (Figure 2B, Figure 2C).

**Figure 2:**
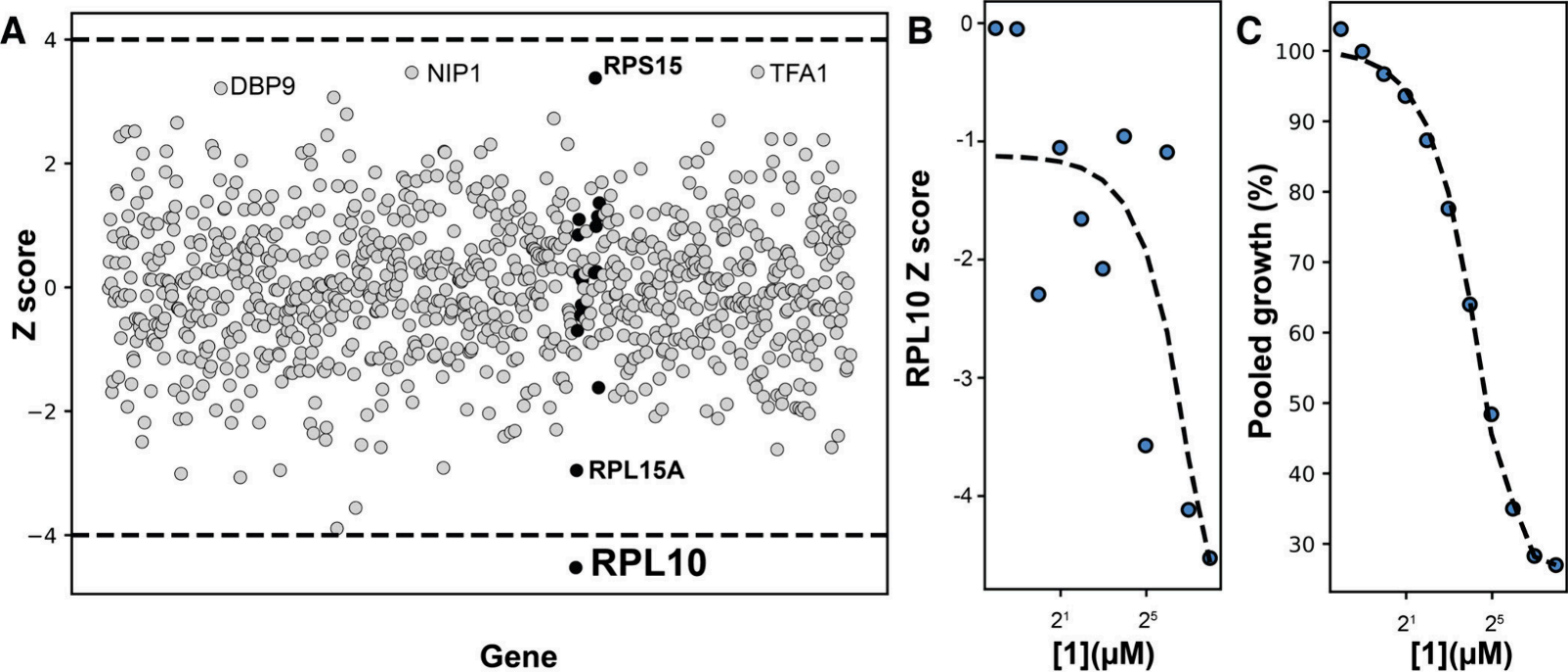
**1** engages RPL10 in S. cerevisiae. (A) Chemical genetic interaction profile of **1**. Horizontal lines depict a significance threshold of Z = ± 4. Ribosomal proteins are highlighted in black (Figure S3) (B) Dose dependence of the RPL10 Z-score of **1** (0.24 μM-250 μM). (C) Dose-dependent growth inhibition of the pool of heterozygous deletion mutants by **1**. In panels (B) and (C), X axes plotted on log_2_ scale and curves represent polynomial fits (*n* = 4).

To complement the S. cerevisiae chemical genetics studies, we pursued an orthogonal approach by analyzing biophysical interactions within the human proteome via Isothermal Shift Assay (iTSA).^31^ In this method, HeLa cells were lysed to produce a protein-rich lysate which was split into two equal pools. The first was treated with 100 μM **1** in DMSO while the second was treated with just DMSO. The lysates were heated to 53 °C, centrifuged, and the soluble portion subjected to LCMS-based bottom-up proteomics. Protein-metabolite engagement can alter the thermal stability of the protein, manifesting as either its decrease (destabilization) or increase (stabilization) in the soluble portion of the treated versus untreated lysate. In agreement with our chemical genetics data RPL10 emerges as a major interaction, showing significant destabilization in this analysis (Figure 3). Among the ribosomal proteins, RPL10 is the only one to show a significant shift in stability, suggesting that the effect is not general to the entire ribosome.

**Figure 3:**
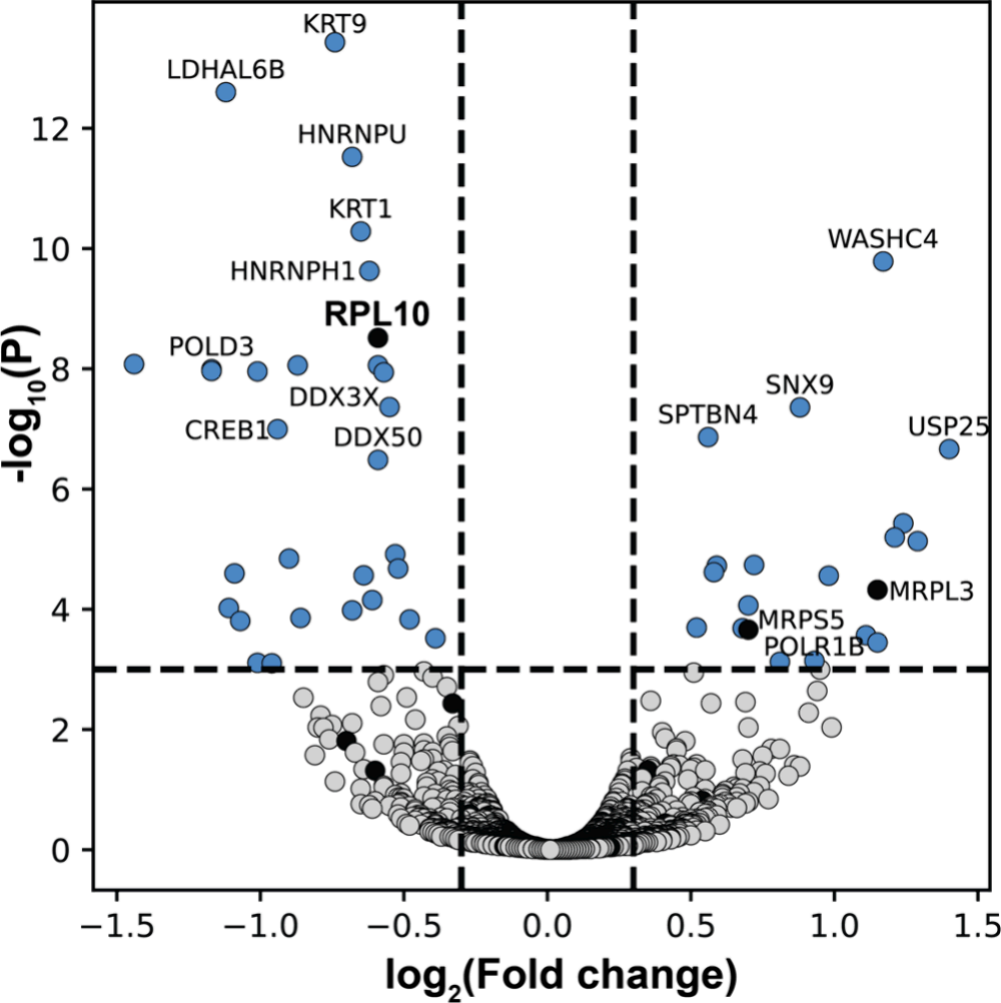
Proteomic isothermal shift assay (iTSA) data for HeLa cell lysates treated with **1** (100 μM). Significant interactions are blue and ribosomal proteins are black.

As RPL10 is instrumental in ribosome function, we evaluated whether **1** could inhibit protein translation. A common method to interrogate protein translation is SUnSET, in which puromycin is incubated with live cells where it is integrated into actively synthesized peptides.^32^ Protein translation inhibitors reduce fluorescence intensity upon treatment with an antipuromycin antibody. Treatment of HeLa cells with 100 μM **1** resulted in reduced puromycin incorporation, suggesting **1** can inhibit protein translation in human cells, albeit with significantly reduced activity as compared to rocaglamide, a known potent translation inhibitor (Figure 4).

**Figure 4:**
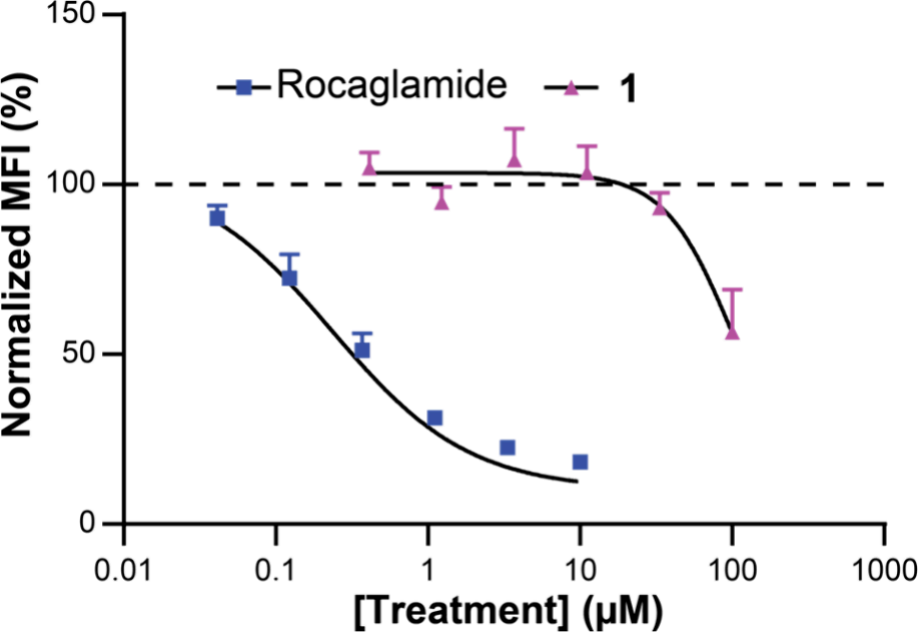
Activity of **1** in a puromycin pulse labeling assay. MFI = mean fluorescence intensity, *n* = 3.

Taken together, the discovery and characterization of the *lig* BGC both elucidates the biosynthesis of **1** and reveals its ability to directly interact with RPL10. Previously known examples of resistance genes encoded within fungal BGCs span a wide range of essential biological processes, including the proteasome, glycolysis, oxidative phosphorylation, as well as the biosyntheses of amino acids, ergosterol, and sphingolipids among others.^9–16^ This work adds ribosomal proteins and protein translation to this list, demonstrating how resistance gene-guided genome mining continues to uncover novel inhibitors of key biological processes. These findings further underscore the vast, untapped potential for discovering new bioactivities hidden within microbial genomes.

## Supplementary Material



## Notes

Supporting data, detailed methods, and NMR spectra (PDF)

## Notes

Proteomic data sets from iTSA experiments are deposited in MassIVE (massive.ucsd.edu, MassIVE id:MSV000096041). The annotated sequence of the *lig*BGC is available in GenBank with accession number PV126210.

## Abbreviations

BGC: biosynthetic gene clusters
HMG-CoA: 3-hydroxy-3-methylglutaryl coenzyme A
NRPKS: nonreducing polyketide synthase
SDR: short-chain dehydrogenase/reductase
MFS: major facilitator superfamily transporter
iTSA: isothermal shift assay
HRMS: high-resolution mass spectrometry
