# Supplementary material for: Resistance Gene-Guided Genome Mining Reveals RPL10 as the Target of Ligustrone A
Source: J Am Chem Soc. 2025 Jun 16;147(25):21284–9. doi: 10.1021/jacs.5c03802 (PMC12203571; doi:10.1021/jacs.5c03802)
Supplement: Supplementary file 1 [file ja5c03802_si_001.pdf]

Supporting information for: Resistance gene-guided genome mining reveals RPL10 as the target of ligustrone A

Danielle A. Yee, Ross D. Overacker, Michelle F. Grau, Amber Cornelius, Adrianne Pigula, Daniel Mummau, Thomas Pavey, Cynthia Bailey, Clarence Hue Lok Yeung, Lawrence S. Hon, Joseph E. Sprak-er, Sheena Li, Bruno Perlatti, Samuel K. Oteng-Pabi, Henry H. Le*, Colin J.B. Harvey*

Supporting Figures **3**

Figure S1: A) lig and bdlig BGCs. Domain architecture of the core NRPKS gene is delineated. SAT = starter unit acyltransferase, KS = ketosynthase, AT = acyltransferase, PT = product template domain, ACP = acylcarrier protein, TE = thioesterase B) Features identified as being related to the lig BGC by untargeted metabolomics of cultures of *A. nidulans ligABCEFGHIKLM* 3

Figure S2: $\log_2$(fold change) in expression of genes in and adjacent to the *lig*BGC in *P. paradoxum* upon overexpression of *ligJ* as compared to control strains containing an empty vector 4

Figure S3: Incubation of 10 μM **1** with 1 mM glutathione (GSH) in PBS buffer for 1 hour leads to spontaneous formation of **10** 5

Figure S4: Incubation of 10 μM **1** with wild type *A. nidulans* leads to formation of **2** in the absence of any genes from the *lig*BGC 5

Figure S5: A) Amino acid alignment of RPL10 from *S. cerevisiae*, *H. sapiens*, *P. paradoxum*, *B. dothidea* and RPL10 homologues LigJ and bdLigJ from the *lig* and *bdlig* BGCs. C_{105} is indicated to highlight its mutation in both putative resistance genes. **B)** alignment of the AlphaFold predicted structures of LigH and *S. cerevisiae* RPL10 shows very strong structural agreement (RMSD = 0.31 Å) **6**

Figure S6: Z-scores for all ribosomal proteins observed in a chemical genetics screen with 250 μM **1**. All ribosomal proteins in the pool are both highlighted black and listed in the table **7**

Supporting Methods **7**

Strains and Fermentation Conditions 7

Media and buffer composition 7

Genome extraction, sequencing, and assembly 8

Plasmid Assembly 8

Transformation of *A. nidulans* and *P. paradoxum* 9

Sample Analysis of *A. nidulans* Transformants and *P. paradoxum* Extracts 9

Metabolomic Mass Spectrometry Analysis 10

Untargeted Metabolomic Analysis 10

Molecular Networking 10

Isolation of Compounds 10

Isothermal Proteome Shift Assay (iTSA) 11

Proteomic Analysis 12

Heterozygous Diploid (HET) Collection 12

Chemical-Genetic Profiling ... 13
Puromycin Labeling Analysis ... 13
Non-enzymatic Glutathione Conjugation ... 13
Aspergillus Nidulans conversion of Ligustrone A (**1**) to B (**2**) ... 13
P. paradoxum ligJ Overexpression RNAseq ... 14
Table S1: Primers used in this study ... 14
Table S2: NMR spectroscopic data of ligustrone A (**1**) ... 19
Table S3: NMR spectroscopic data of ligustrone B (**2**) ... 20
Table S4: NMR spectroscopic data of topopyrone C (**4**) ... 22
Table S5: NMR spectroscopic data of **6** ... 23
Table S6: NMR spectroscopic data of **8** ... 24
Table S7: NMR spectroscopic data of **9** ... 26
Table S8: NMR spectroscopic data of **10** ... 27
NMR Spectra ... 29
Figure S7: ^{1}H NMR Spectrum of ligustrone A (**1**) (600MHz, DMSO-d_6) ... 29
Figure S8: ^{13}C NMR Spectrum of ligustrone A (**1**) (150MHz, DMSO-d_6) ... 29
Figure S9: ^{1}H NMR Spectrum of ligustrone B (**2**) (600MHz, CD_3CN) ... 30
Figure S10: ^{13}C NMR Spectrum of ligustrone B (**2**) (150MHz, CD_3CN) ... 30
Figure S11: ^{1}H NMR Spectrum of topopyrone C (**4**) (600MHz, acetone-d_6) ... 31
Figure S12: ^{13}C NMR Spectrum of topopyrone C (**4**) (150MHz, acetone-d_6) ... 31
Figure S13: ^{1}H NMR Spectrum of **6** (600MHz, DMSO-d_6) ... 32
Figure S14: ^{13}C NMR Spectrum of **6** (150MHz, DMSO-d_6) ... 32
Figure S15: ^{1}H-^{13}C HSQC NMR Spectrum of **6** (600MHz, DMSO-d_6) ... 33
Figure S16: ^{1}H-^{13}C HMBC NMR Spectrum of **6** (600MHz, DMSO-d_6) ... 33
Figure S17: ^{1}H NMR Spectrum of **8** (600MHz, CD_3CN) ... 34
Figure S18: ^{13}C NMR Spectrum of **8** (150MHz, CD_3CN) ... 34
Figure S19: ^{1}H-^{1}H COSY NMR Spectrum of **8** (600MHz, CD_3CN) ... 35
Figure S20: ^{1}H-^{13}C HSQC NMR Spectrum of **8** (600MHz, CD_3CN) ... 35
Figure S21: ^{1}H-^{13}C HMBC NMR Spectrum of **8** (600MHz, CD_3CN) ... 36
Figure S22: ^{1}H NMR Spectrum of **9** (600MHz, CD_3CN) ... 36
Figure S23: ^{13}C NMR Spectrum of **9** (150MHz, CD_3CN) ... 37
Figure S24: ^{1}H-^{1}H COSY NMR Spectrum of **9** (600MHz, CD_3CN) ... 37
Figure S25: ^{1}H-^{13}C HSQC NMR Spectrum of **9** (600MHz, CD_3CN) ... 38
Figure S26: ^{1}H-^{13}C HMBC NMR Spectrum of **9** (600MHz, CD_3CN) ... 38
Figure S27: ^{1}H NMR Spectrum of **10** (600MHz, CD_3OD) ... 39
Figure S28: ^{13}C NMR Spectrum of **10** (150MHz, CD_3OD) ... 39
Figure S29: ^{1}H-^{1}H COSY NMR Spectrum of **10** (600MHz, CD_3OD) ... 40
Figure S30: ^{1}H-^{13}C HSQC NMR Spectrum of **10** (600MHz, CD_3OD) ... 40
Figure S31: ^{1}H-^{13}C HMBC NMR Spectrum of **10** (600MHz, CD_3OD) ... 41
References ... 41

Supporting Figures

A

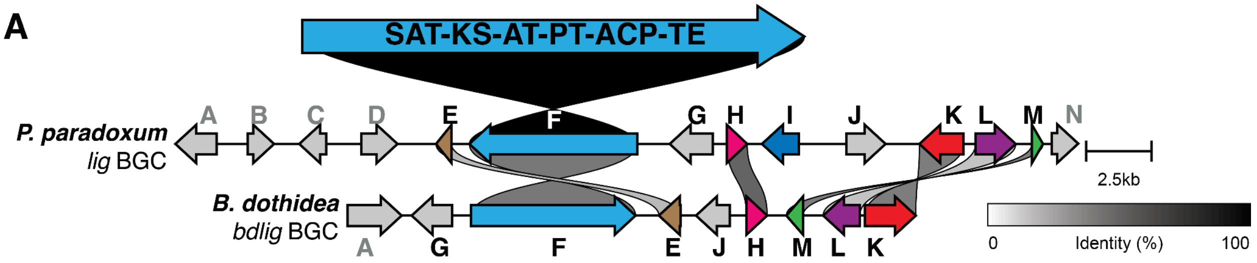

B

	m/z (ESI+)	Formula $[M+H]^+$
Polyketide (PK)	301.071	$C_{16}H_{13}O_6$
	319.081	$C_{16}H_{15}O_7$
	313.071	$C_{17}H_{13}O_6$
	317.102	$C_{17}H_{17}O_6$
	339.050	$C_{18}H_{11}O_7$
	309.075	$C_{18}H_{13}O_5$
	311.091	$C_{18}H_{15}O_5$
	341.102	$C_{19}H_{17}O_6$
	577.076	$C_{32}H_{17}O_{11}$
	559.138	$C_{34}H_{23}O_8$
PK + Sulfur	369.042	$C_{19}H_{13}O_6S$
	401.069	$C_{20}H_{17}O_7S$
	429.063	$C_{21}H_{17}O_8S$
	431.081	$C_{21}H_{19}O_8S$
	616.159	$C_{28}H_{30}N_3O_{11}S$

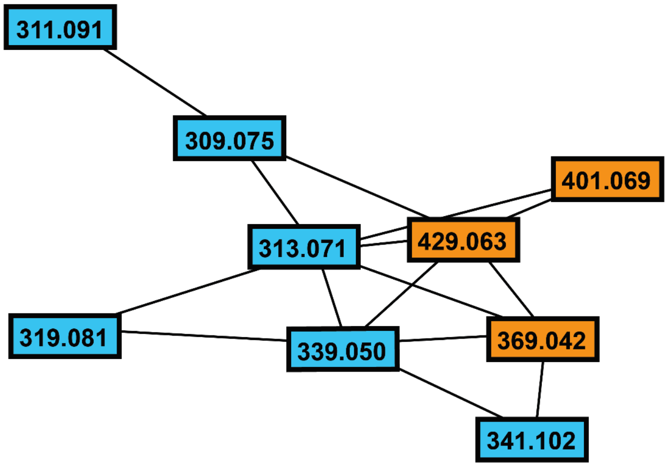

Figure S1: A) *lig* and *bdlig* BGCs. Domain architecture of the core NRPKS gene is delineated. SAT = starter unit acyltransferase, KS = ketosynthase, AT = acyltransferase, PT = product template domain, ACP = acylcarrier protein, TE = thioesterase **B)** Features identified as being related to the *lig* BGC by untargeted metabolomics of cultures of *A. nidulans ligABCEFGHIKLM.*

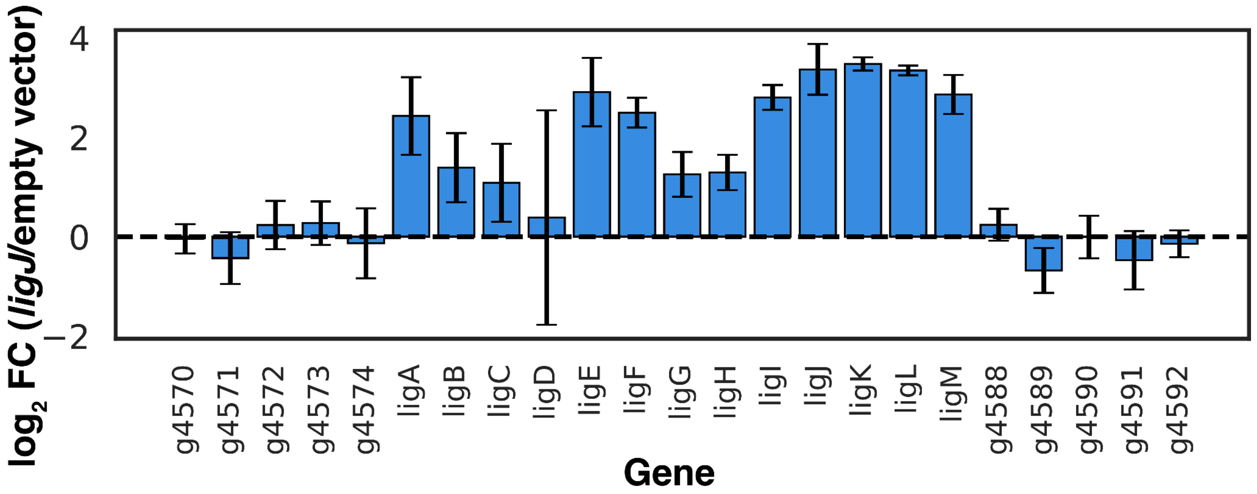

Figure S2: log$_2$(fold change) in expression of genes in and adjacent to the *lig*BGC in *P. paradoxum* upon overexpression of *lig*J as compared to control strains containing an empty vector.

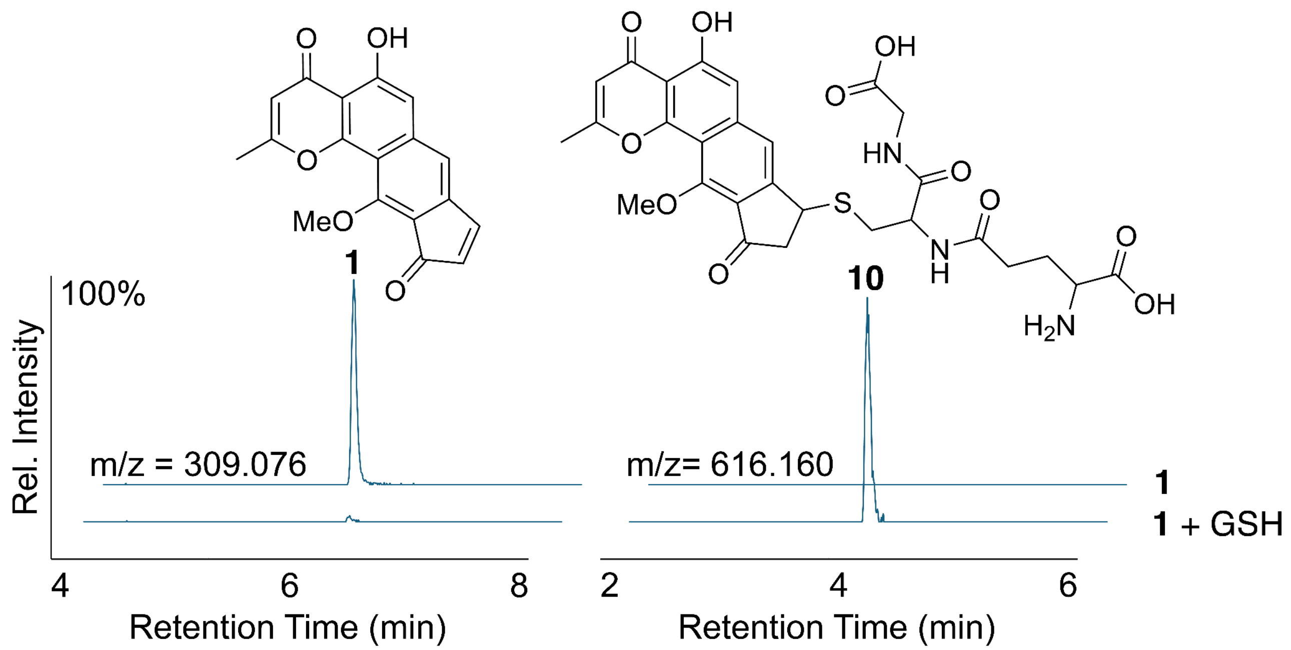

Figure S3: Incubation of 10 μM **1** with 1 mM glutathione (GSH) in PBS buffer for 1 hour leads to spontaneous formation of **10**.

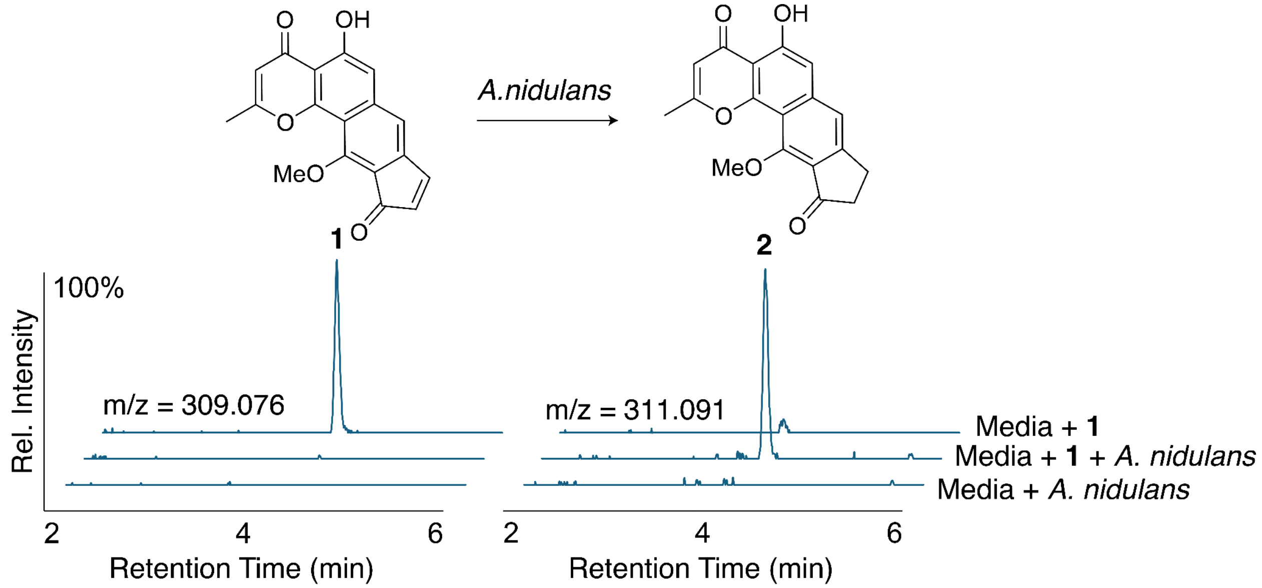

Figure S4: Incubation of 10 μM **1** with wild type *A. nidulans* leads to formation of **2** in the absence of any genes from the *lig*BGC.

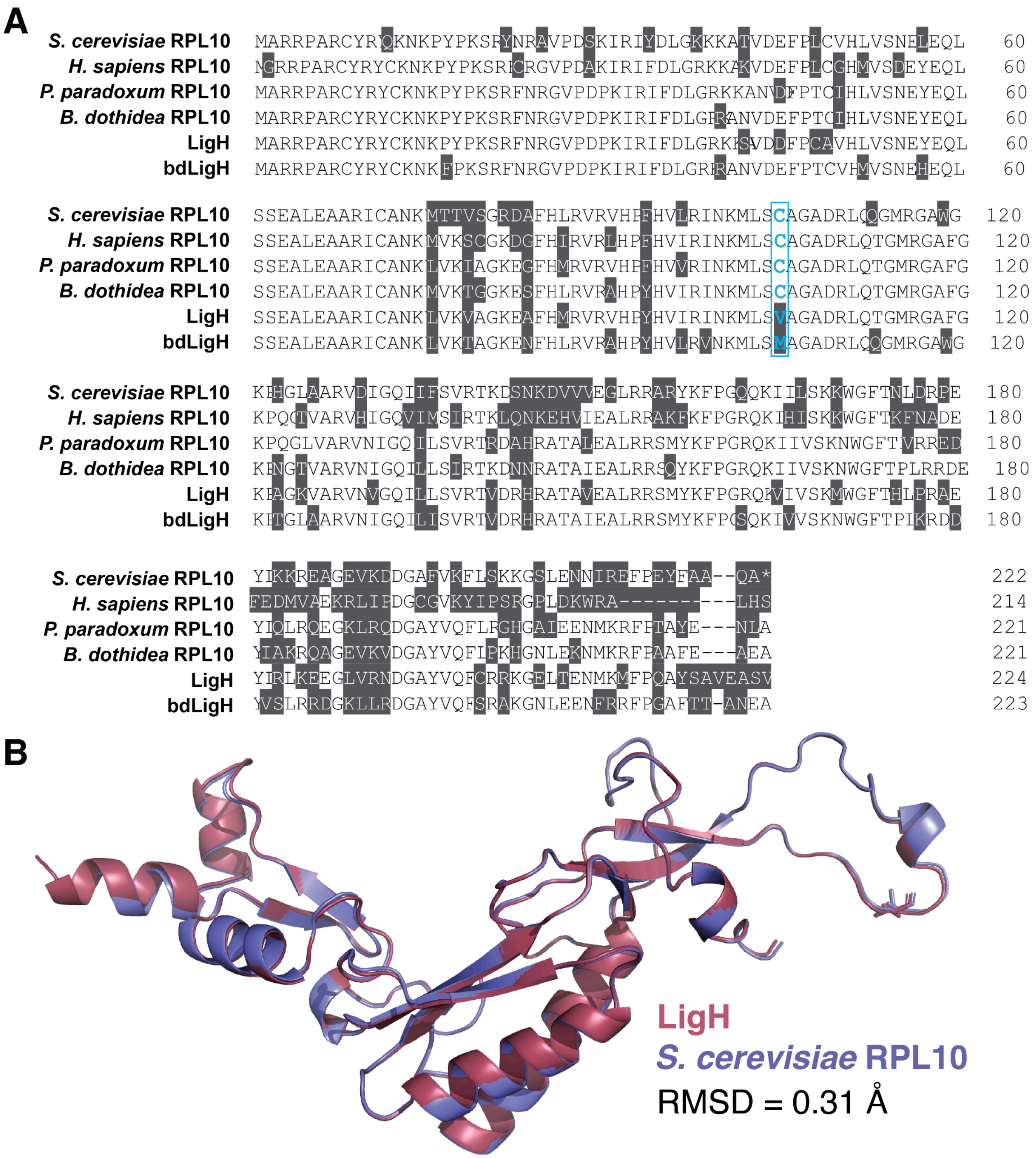

Figure S5: A) Amino acid alignment of RPL10 from *S. cerevisiae*, *H. sapiens*, *P. paradoxum, B. dothidea* and RPL10 homologues LigJ and bdLigJ from the lig and bdlig BGCs. C105 is indicated to highlight its mutation in both putative resistance genes. **B)** alignment of the AlphaFold predicted structures of LigH and *S. cerevisiae* RPL10 shows very strong structural agreement (RMSD = 0.31 Å)

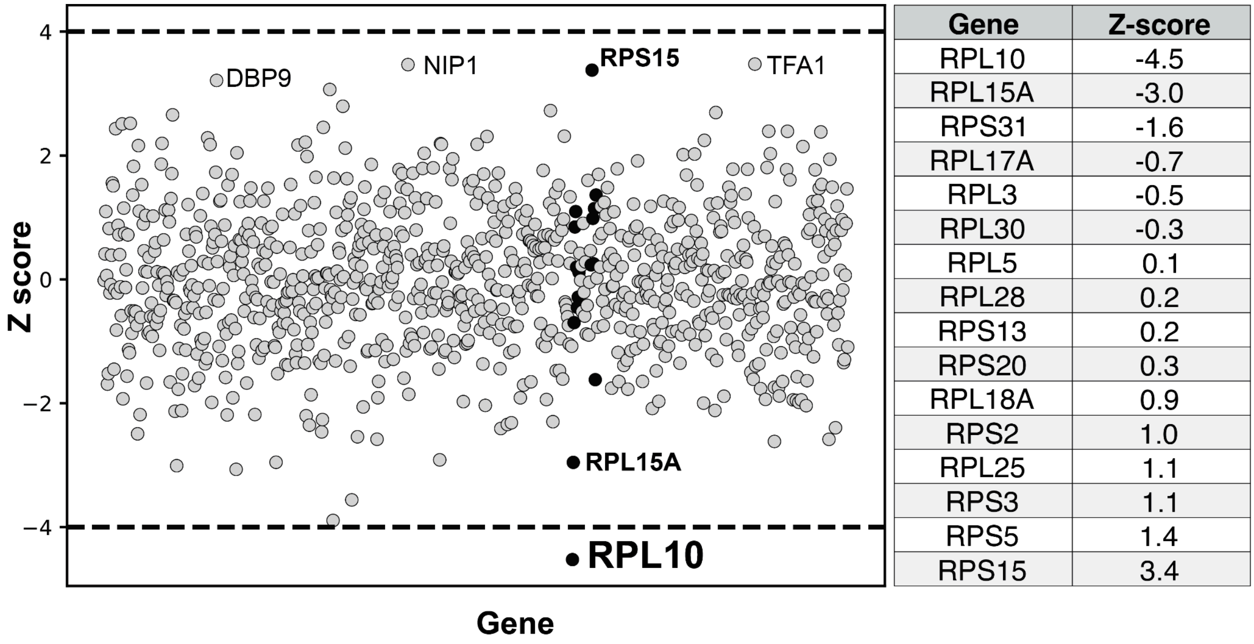

Gene	Z-score
RPL10	-4.5
RPL15A	-3.0
RPS31	-1.6
RPL17A	-0.7
RPL3	-0.5
RPL30	-0.3
RPL5	0.1
RPL28	0.2
RPS13	0.2
RPS20	0.3
RPL18A	0.9
RPS2	1.0
RPL25	1.1
RPS3	1.1
RPS5	1.4
RPS15	3.4

Figure S6: Z-scores for all ribosomal proteins observed in a chemical genetics screen with 250 µM **1**. All ribosomal proteins in the pool are both highlighted black and listed in the table.

Supporting Methods

Strains and Fermentation Conditions

Penicillium paradoxum (NRRL2162, Country of origin: New Zealand) was obtained from the Agricultural Research Service Culture Collection (NRRL) fungal collection. *P. paradoxum* was grown on Difco™ PDA (Potato Dextrose Agar) plates from BD biosciences for maintenance culturing. BgHX27 (pyrG89; pyroA4; nkuA::argB; riboB2; stc::AfpyrG; AN1036-AN1028::AfriboB) is a modified version of *A. nidulans* A1145 purchased from the Fungal Genetics Stock Center and was used for heterologous expression of genes from *P. paradoxum*. *Escherichia coli* strain DH10B was used for cloning. *Saccharomyces cerevisiae* strain JHY692 (MATa his3Δ1 leu2Δ0 ura3Δ0 met15Δ0 SAL1+ HAP1+ CAT5(91M) MIP1(661T) MKT1(30G) RME1(INS- 308A) TAO3(1493Q) prb1Δ pep4ΔADH2p-npgA-ACS1)[1] was used for homologous recombination of DNA fragments to assemble the vectors used in heterologous expression.

Media and buffer composition

ST buffer: 1.2 M sorbitol, 12.5 mM Tris-HCl, pH 7.5

STC buffer: 1.2 M sorbitol, 10 mM calcium chloride, 12.5 mM Tris-HCl, pH 7.5

SMM: 1.2 M sorbitol, 10 g/L glucose, 6 g/L $NaNO_3$, 0.52 g/L KCl, 0.52 g/L $MgSO_4 \cdot 7H_2O$, 1.52 g/L KH_2PO_4, 1 mL/L trace elements concentrate, 0.05 w/v Riboflavin

CYA: 30 g/L sucrose, 5 g/L yeast extract, 1.3 g/L potassium phosphate dibasic, 3 g/L sodium nitrate, 10 mL/L Czapek concentrate

GMM: 10 g/L glucose, 6 g/L $NaNO_3$, 0.52 g/L KCl, 0.52 g/L $MgSO_4 \cdot 7H_2O$, 1.52 g/L KH_2PO_4, 1 mL/L trace elements concentrate, 0.05 w/v Riboflavin

M102: 30 g/L sucrose, 20 g/L malt extract, 1 g/L yeast extract, 2 g/L peptone, 0.5 g/L K_2HPO_4, 0.5 g/L $MgSO_4 \cdot 7H_2O$, 0.5 g/L KCl, pH 6

Medium I: 20 g/L glucose, 5 g/L peptone, 1 g/L KH_2PO_4, 0.5 g/L $MgSO_4 \cdot 7H_2O$, 0.5 g/L KCl, 10 mg/L $FeSO_4$.7H2O, 3 g/L $NaNO_3$

MEY: 20 g/L malt extract, 5 g/L yeast extract

MGTY: 15 g/L maltose, 10 g/L yeast extract, 10 g/L tryptone, 1 g/L KH_2PO_4, 0.2 g/L $MgSO_4$·7H2O, 0.5 g/L $CaCl_2.2H_2O$, 10 g/L glycerol

MME: 30 g/L malt extract, 3 g/L peptone, 10 mg/L $ZnSO_4.7H_2O$, 5mg/L $CuSO_4 \cdot 5H_2O$

MMSY: 40 g/L mannitol, 5 g/L yeast extract, 4.3 g/L Murashige and Skoog Basal Salt Mixture

SMK: 40 g/L soluble starch, 1 g/L yeast extract, 4.3 g/L Murashige and Skoog Basal Salt Mixture

Supermalt: 50 g/L malt extract, 10 g/L yeast extract, 20 mg/L $FeSO_4.7H_2O$, 7 mg/L $ZnSO_4.7H_2O$)
WATM (30 g/L sucrose, 2 g/L yeast extract, 3 g/L peptone, 1 g/L K_2HPO_4, 0.2 g/L KCl, 50 mg/L $FeSO_4.7H_2O$, 2 g/L $NaNO_3$, 10 mg/L $ZnSO_4.7H_2O$, 5 mg/L $CuSO_4 \cdot 5H_2O$, 5 g/L corn steep solids

YPSS: 14 g/L soluble starch, 4 g/L yeast extract, 1 g/L K_2HPO_4, 0.5 g/L $MgSO_4 \cdot 7H_2O$

Trace elements concentrate: 1.0 g/L $FeSO_4.7H_2O$, 1.0 g/L $MnSO_4.H_2O$, 0.2 g/L $ZnSO_4.7H_2O$, 0.1 g/L $CaCl_2.2H_2O$, 0.056 g/L HBO_3, 0.025 g/L $CuCl_2.2H_2O$, 0.019 g/L $(NH_4)6.Mo_7O_{24}.4H_2O$, 50 mL/L 12M HCl)

Genome extraction, sequencing, and assembly.

Strains were cultured on malt and yeast extract (MEYA) medium consisting of 2% w/v malt extract, 0.5% w/v yeast extract and 0.5% w/v agar at 25 °C for 14 days. Mycelium was harvested, frozen at -80 °C, lyophilized, and pulverized via bead beating. Genomic DNA was purified from ground mycelial powder with a Zymobiomics MagBead DNA/RNA (Zymo Research, United States). For preparation of sequencing libraries, ~100 ng of total genomic DNA was processed using the KAPA HyperPlus Kit for PCR-free workflows (Roche, Switzerland), followed by seven rounds of amplification to increase library yields. Sequencing libraries were pooled and size-selected for 300-800 bp fragments using a Qiagen GeneRead Size Selection Kit (Qiagen, Germany). Sequencing was performed by Admera Health (South Plainfield, NJ) on a NovaSeq 6000 System (Illumina, USA) to obtain 118M paired-end reads of 151bp (18Gb). Genome assembly was performed with SPAdes v3.15.5[2] (https://github.com/ablab/spades). The benchmarking universal single-copy orthologs (BUSCO v3.0.2) tool was used to evaluate the integrity of the genome assembly. The genomes were annotated with the BRAKER2 platform[3–5] (https://github.com/Gaius-Augustus/BRAKER) using the AUGUSTUS 3.3.2 package.

Plasmid Assembly

Plasmids containing donor DNA for genomic integrations were constructed as described

previously[6]. Plasmids pHex383 and pHex384 were used as backbone vectors for plasmid assembly. pHex383 contains a PacI site flanked by the *A. nidulans* 1036p and 1034p promoters. pHex384 contains a PacI site flanked by AN1034p and AN1029p. pHex384 also contains the *A. nidulans* transcription factor AN1029 under the alcA promoter, AN1028p, a PmeI cut site, the auxotrophic marker for pyridoxine (pyroA), and AN1028t downstream of AN1029p. The homology regions on both pHex383 and pHex384 are flanked by SceI sites. All genes from *P. paradoxum* were amplified by PCR using *P. paradoxum* genomic DNA as the template with primers defined in Table S1. pHex383 and pHex384 were linearized by digestion with PacI and were co-transformed with their respective gene fragments into JHY692 to assemble pDY0065 and pDY0066 through yeast homologous recombination. To generate pDY0067, ligI with its native terminator was amplified by PCR using *P. paradoxum* genomic DNA as the template. pDY0066 was linearized by digestion with PmeI. ligI was cloned into pDY0066 using NEBuilder HiFi DNA Assembly. The resulting plasmids were verified by sequencing and digested with SceI to generate linear donor DNA. For pDY0068, the transcription factor ligJ was amplified by PCR using *P. paradoxum* genomic DNA as the template. ligJ was cloned under the constitutive promoter, PcoxAN in the pHex385 backbone. The pHex385 backbone contains a hygromycin selectable marker (hph) and the AMA1 fungal origin of replication.

Transformation of *A. nidulans* and *P. paradoxum*

Spores of *A. nidulans* were inoculated into 25 mL YG medium and incubated in stationary culture at 37 °C overnight. For *P. paradoxum*, spores were inoculated into 25 mL supermalt medium and incubated in stationary culture at room temperature for 2 days. The resulting biomass was filtered using a sterile cell strainer (Fisher, Catalog No. 22363547), washed with 10 mL of Phosphate Buffered Saline (PBS, Gibco Cat# 10-010-049), and then transferred into 25 mL of digestion buffer containing 3 g of VinoTaste® Pro (Novozymes) and 100 mg of Yatalase™ Enzyme (Takara Bio Catalog No. T017). The germlings were incubated for 2 h at 30 °C and 80 rpm. The digestion buffer for *A. nidulans* consisted of $MgSO_4.7H_2O$ (14.79g) and 175 mM sodium phosphate buffer (up to 50 mL). The digestion buffer for *P. paradoxum* consisted of KCl (1.1 M) and citric acid monohydrate (0.1 M), pH 5.8. The digested *A. nidulans* cells were filtered through a cell strainer into a 50-mL conical tube and centrifuged at 1,800 × g for 15 min. The protoplasts were washed with 0.6 M KCl, collected by centrifugation (1,800 × g for 10 min), and resuspended in STC buffer prior to transformation. For *P. paradoxum*, the digestion mix was poured into a 50-mL conical tube and overlaid with 10 mL of 0.4 M ST Buffer and centrifuged at 1,800 × g for 15 min. The protoplasts were then removed from the interface of the two buffers and transferred to sterile tubes. The protoplasts were washed with 0.6 M KCl, collected by centrifugation (1,800 × g for 10 min), and resuspended in STC buffer. For both strains, DNA was added to the transformation mix and incubated on ice for an hour. PEG solution (60% w/v PEG 3350, 50 mM $CaCl_2$, and 50 mM Tris- HCl) was added to the protoplast solution and incubated at room temperature for 20 min. The cells were then plated onto solid SMM (with the addition of 0.125 µg/mL riboflavin for *A. nidulans*). For *P. paradoxum*, the next day an equal volume of SMM was overlaid over each plate containing hygromycin to a final concentration of 30 µg/mL. After transformants appeared on the plates, the *A. nidulans* spores were restreaked onto solid GMM medium supplemented with riboflavin at 37 °C for 3 d. The spores were collected with 0.01% Tween 80 solution and transferred into liquid LMM media for metabolite production.

Sample Analysis of *A. nidulans* Transformants and *P. paradoxum* Extracts

A. nidulans cultures were grown in liquid LMM at 30 °C shaking at 180 rpm. After two days, gene expression was induced by addition of 2-butanone (50 mM). The cultures were then grown at 30 °C shaking at 180 rpm for 3 additional days. *P. paradoxum* strains were grown in a panel

of 12 growth media (See 'Media Composition' above). Strains were grown in 96 well deep well plates with 500 µL of media. The plates were then frozen and lyophilized. The samples were then resuspended in 1:1 MeOH and injected onto the LC-MS for analysis.

Metabolomic Mass Spectrometry Analysis

High-resolution LCMS analysis was performed on a Thermo Fisher Scientific Vanquish Horizon UHPLC System coupled with a Thermo Q Exactive hybrid quadrupole-orbitrap high-resolution mass spectrometer equipped with a HESI ion source. Two µL of extract was injected and separated using a water-acetonitrile gradient on an Accucore Vanquish C18+ Reversed Phase HPLC Column (1.5 µm, 2.1 mm x 50 mm, Cat# 27101-052130) maintained at 50 ℃. Solvent A: 0.1% formic acid in water; Solvent B: 0.1% formic acid in acetonitrile. A/B gradient started at 12.5% B increased linearly to 97% B at 3.5 min and held at 97% B for 1 min, using a flow rate 0.6 mL/min. Mass spectrometer parameters: spray voltage 3 kV for positive mode, capillary temperature 380 ℃, prober heater temperature 400 ℃; sheath, auxiliary, and spare gas 60, 20, and 1, respectively; S-lens RF level 50, resolution 140,000 at m/z 200, AGC target 3E10. Tandem mass spectrum analysis was carried out with an exclusion list of known endogenous features with the same parameters (*vide supra*) with the following additions or adjustments: Full MS Resolution: 35,000, AGC target 1E6, Max IT: 30 ms, scan range: 150 to 1400 m/z, ddMS2 Resolution: 17,500, AGC target 5E4, Max IT: 50 ms, Loop Count: 10, isolation window: 2.0 m/z, stepped collision energy: 20, 40, 60 NCE.

Untargeted Metabolomic Analysis

RAW files generated from HPLC-HRMS acquisitions were converted to mzXML files utilizing MSconvertGUI software (proteowizard.sourceforge.net). Differential metabolic features were determined by subjecting mzXML files to Metaboseek Software version 0.9.9[7] (metaboseek.com) utilizing the XCMS package with all values set to default parameters which are as follows: Peak Detection: ppm 4, peakwidth 320, snthresh 3, prefilter 3100, fitgauss FALSE, integrate 1, firstBaselineCheck TRUE, noise 0, mzCenterFun wMean, mzdiff -0.005, workers 4. Peak filling: METABOSEEK, ppm_m 5, rtw 5, rtrange TRUE, areaMode FALSE. Feature Grouping: minfrac 0.2, bw 2, mzwid 0.002, max 500, minsamp 1, usegroups FALSE. CAMERA and RT correction were not used. Differential molecular features were sorted using the minFoldOverCtrl, minInt, and Fast_Peak_Quality filters. Differential features were subjected to manual curation to remove adducts, isotopes, and false positives. The curated list of molecular features were assigned molecular formulas and then subjected to tandem mass spectrometry for further elucidation. LC-MS files have been deposited to the GNPS Web site (massive.ucsd.edu) with MassIVE ID numbers MSV000096041.

Molecular Networking

Using an LCMS sample that contained spectra for the relevant m/zs, the highest intensity spectra for each m/z was identified. We used MS2DeepScore[8] to convert spectra into embeddings, which can be thought of as neural network learned spectral fingerprints that facilitate spectral comparisons. The converted embeddings were then compared using cosine similarity, and spectra with MS2DeepScore similarity >0.7 were plotted with edges in a molecular network using NetworkX.

Isolation of Compounds

24 l cultures were extracted using a mixture of HP20/and XAD-7 resins. Ten g of a 1:1 mixture

of resins was washed with methanol and water then heat sealed into tea bags. One tea bag per 1 L culture was allowed to shake with culture for 3 hours in the culturing incubator. Resin bags were removed and combined followed by extraction using excess volumes of ethyl acetate and methanol, sequentially. Organic crude extract was collected and dried over an excess of anhydrous magnesium sulfate followed by filtration and concentration *in vacuo*. Crude extract was dry-loaded onto Celite and subjected to fractionation using a Biotage Selekt with a 120 g C18 column (Catalog No. FSUD-0401-0120) following a gradient of 0.1% aqueous formic acid (A) and acetonitrile with 0.1% formic acid (B) at 40 mL/min (20%B for 1 CV, 20 - 100%B for 7 CV, 100%B for 3CV). Forty eight fractions were collected and analyzed *via* liquid chromatography-high resolution mass spectrometry (LC-HRMS) using 0.1% aqueous formic acid (A)/acetonitrile with 0.1% formic acid (B) with a linear gradient of 12.5 to 100% B in 5 min, and desired fractions were pooled. They were further fractionated using a semipreparative C18 column (XBridge BEH Shield RP18 OBD, 10 × 250 mm, 10 μm) with gradient method (35% for 3 min; 35 to 75% in 41 min; 75 to 100% in 1 min; hold 100% for 6 min) using the same solvents as above, at 4 mL/min, yielding **1** (r.t. 14.2 min, 8.0 mg), **2** (r.t. 36.8 min, 4.2 mg), **4** (r.t. 27.2 min, 1.4 mg), **6** (r.t. 23.7 min, 1.2 mg), **8** (r.t. 17.8 min, 2.2 mg), **9** (r.t. 7.7 min, 2.3 mg), and **10** (r.t. 16.2 min, 2.0 mg).

Isothermal Proteome Shift Assay (iTSA)

HeLa (ATCC CCL-2) cells were maintained in Eagle's minimum essential medium (EMEM) containing 10% FBS and 1x Penicillin-Streptomycin. Three million cells were harvested with a cell scraper and centrifuged, washed with PBS, and then flash frozen with liquid nitrogen and stored at -80 ℃ until further processing. Frozen cells were thawed and then suspended in ice-cold PBS containing 1x Pierce Protease Inhibitor Tablets (ThermoFisher Cat# A32963). The cells were then sonicated for 2 minutes in a cup horn sonicator filled with ice-cold water. The lysate was then centrifuged for 20 minutes at 20,000 G at 4 ℃. The supernatant was transferred to a fresh, ice-cold centrifuge tube and clarified lysates were quantified via Bradford protein quantification and then diluted to 1 mg/mL. Lysate was split into two equal volumes, and then lysates were treated with either DMSO vehicle or 100 μM **1**. The treated lysates were incubated at 25 ℃ for 30 minutes. Samples were then aliquoted in 100 uL aliquots into PCR tubes and heated to their corresponding temperatures for 3 minutes. After heating, the samples were immediately moved back to ice. Samples were then centrifuged at 20,000 G, 4 ℃, for 20 minutes. The remaining soluble protein was transferred to a fresh tube. Proteins were precipitated by adding 400 μL of ethanol and incubating the samples for 12 hours at -20 ℃. Tubes were then centrifuged at 20,000 G, 4 ℃, for 20 minutes. The ethanolic liquid was carefully removed, and the protein pellet was washed with 500 μL of LCMS-grade methanol. The protein pellet was then re-hydrated with 50 μL of 8 M urea containing 5 mM DTT (Sigma Aldrich Cat# 20-26) and then heated to 37 ℃ for 1 hour. The samples were cooled to 25 ℃ and then 4 μL of 100 mM iodoacetamide (Sigma Aldrich Cat# I1149) was added. The tubes were vortexed, drawn down, and then incubated for 1 hour in the dark. Next, 750 μL of 50 mM ammonium bicarbonate (Sigma Aldrich Cat# 5330050050) containing 5 mM DTT was added, and after brief shaking to mix the solution, the solution incubated for another 1 hour at 25 ℃ to neutralize the remaining iodoacetamide. Proteins were then digested with 1 μg Trypsin/Lys-C Mix, Mass Spec Grade (Promega Cat# V5071) for 18 hours at 37 ℃ on an orbital shaker shaking at 700 RPM. A second pulse of 0.2 μg Trypsin/Lys-C Mix was added, and the sample was incubated (as above) on the shaking incubator for an additional 4 hours. Ten μL of trifluoroacetic acid (ThermoFisher Scientific Cat# 85183) was added. Peptides were then isolated with a Sep-Pak C18 96-well Plate (Waters Cat# 186003966). All Sep-Pak centrifugation (wash) steps will occur at 500 G for 1 minute at 25 ℃. Briefly, the plate was primed with 800 μL of methanol and centrifuged. Then the Sep-Pak was washed with 80% acetonitrile (Fisher

Scientific Cat# A996-4) in water (Fisher Scientific Cat# W7-4) containing 0.1% formic acid (Fisher Scientific Cat# A117-50). The Sep-Pak was washed twice with 800 µL water containing 0.1% formic acid. The samples were applied and centrifuged. The Sep-Pak was, again, washed twice with 800 µL water containing 0.1% formic acid. Peptides were finally eluted with 650 µL of 60% acetonitrile in water containing 0.1% formic acid into Protein LoBind Plates (Eppendorf Cat# 0030504305). The cleaned peptides were dried with a centrifugal evaporator, and peptides were re-suspended in a 100 µL solution of 5% acetonitrile containing 0.1% formic acid. The samples were filtered through a MultiScreen Solvinert 96 Well Filter Plate (Millipore Sigma Cat# MSRLN0410) before LCMS injection.

Proteomic Analysis

High-resolution LC-MS analysis was performed on a Thermo Fisher Scientific Vanquish Neo UHPLC System coupled with a Thermo Exploris 480 hybrid quadrupole-orbitrap high-resolution mass spectrometer equipped with an EASY-Spray Source. Two µL of sample was injected and separated using a water-acetonitrile gradient on a Easy-Spray PepMap Neo C18 (2 µm, 75 µm X 150 mm part number: ES75150PN) maintained at 40 ℃. Solvent A: 0.1% formic acid in water; Solvent B: 0.1% formic acid in acetonitrile. A/B gradient started at 2% B for 2 min after injection and increased linearly to 22% B for 62 min, increased linearly again to 35% B for 12 min, and then immediately increased and held at 100% B for 6 min, using a flow rate 300 nL/min. Mass spectrometer parameters: spray voltage 1.8 kV in positive mode, capillary temperature 320 ℃. The instrument was operated in ddMS2 mode with a cycle time of 3 seconds. Full scan parameters were resolution 120,000, scan range 375-1500 m/z, RF lens 60%, MIPS peptide mode, intensity threshold 8E3, charge state 2-6, dynamic exclusion custom mode, exclude n=1 times, exclusion duration 30 sec, mass tolerance low 10 high 10, exclusion isotopes, MS^2 isolation windows 2 m/z, collision energy normalized HCD 30%, resolution 15000, defined first mass 120. The instrument was calibrated with positive and negative mode using the Pierce FlexMix Calibration Solution (Thermo-Fisher). RAW files were loaded in to Proteome Discoverer 3.0 (Thermo Scientific) and processed with the processing workflow "PWF_Hybrid_LFQ_CHIMERYS" and the consensus workflow "CWF_Comprehensive_Enhanced Annotation_LFQ_and_Precursor_Quan". The UniProt ID UP000005640 was loaded and processing occurred with default parameters. Low confidence values were removed and volcano plots were generated with the VolcaNoseR (huygens.science.uva.nl/VolcaNoseR/) tool. Gene symbols of significantly down-regulated proteins from the isothermal proteome shift analysis were analyzed with Enrichr[8,9] (maayanlab.cloud/Enrichr/). LC-MS files have been deposited to the GNPS Web site (massive.ucsd.edu) with MassIVE ID numbers MSV000097030.

Heterozygous Diploid (HET) Collection

A set of 968 barcoded diploid heterozygous mutants, each containing a heterozygous deletion of a unique essential gene, was constructed in yeast strain Y14486 MATa/MATα pdr1Δ::natMX/pdr1Δ::natMX pdr3Δ::KI.URA3/pdr3Δ::KI.URA3 snq2Δ::KI.LEU2/snq2Δ::KI.LEU2 his3Δ1/his3Δ1 leu2Δ0/leu2Δ0 ura3Δ0/ura3Δ0 met15Δ0/met15Δ0. Oligonucleotide primers were designed to generate kanMX-marked deletions of essential genes, and incorporate 20 bp barcodes upstream of each marked deletion. Diploid transformants were selected on YPD (1% yeast extract, 2% peptone, 2% glucose) media containing G418 (200 µg/mL), and the presence of desired deletions was confirmed by PCR. If we delete a given gene XXX, the resultant heterozygous diploid genotype would be MATa/MATα xxxΔ::kanMX/XXX snq2Δ::KI.LEU2/snq2Δ::KI.LEU2 pdr3Δ::KI.URA3/pdr3Δ::KI.URA pdr1Δ::hygB/pdr1Δ::hygB can1Δ::STE2pr-Sp_his5/can1Δ::STE2pr-Sp_his5 lyp1Δ/lyp1Δ his3Δ1/his3Δ1 leu2Δ0/leu2Δ0

ura3Δ0/ura3Δ0 met15Δ0/met15Δ0.

Chemical-Genetic Profiling

Pooled yeast mutant libraries were treated with **1** as previously described[10]. Libraries were each pooled and distributed into separate wells in 96-well plates. Each well contained 4.65×105/mL cells, 196 µL YPGal medium (1% yeast extract, 2% peptone, 2% galactose) and 2 µL compound at different concentrations. OD_{600} was measured after 24 h using a plate reader, at which point the percentage of growth inhibition relative to the DMSO-only control (1% DMSO) was calculated to measure cell growth. This was followed by incubation for an additional 24 h at 30 °C. The plates were then centrifuged at 3,000 rpm for 4 min before adding 125 µL zymolyase dissolved in 1 M D-sorbitol (final concentration: 0.5 mg/mL) with 11.5 µM β-mercaptoethanol. The samples were incubated for 1 h at 37 °C, centrifuged again at 4,000 rpm for 5 min and processed using the QIAamp 96 DNA kit. Genomic DNA extraction was performed with an automated high-throughput nucleic acid purification robot, QIAcube HT (QIAGEN). Strain-specific DNA barcodes were amplified using multiplex primers and a communal U2 primer. PCR conditions were set as follows: 3 min at 95 °C for initial denaturation, 30 cycles of 15 s at 95 °C, 15 s at 60 °C, 20 s at 72 °C, and a final extension time of 5 min at 72 °C. PCR products were purified from 2% agarose gels using a Geneclean III kit, quantified using a Kapa qPCR kit and sequenced with an Illumina Hiseq 2500.

Puromycin Labeling Analysis

The SUnSET assay was run as described previously.[11] HeLa cells were cultured and maintained in Dulbecco's Modified Eagle Medium (DMEM) supplemented with 10% FBS. Cells were seeded at a density of 5000 cells per well in a 96 well plate and allowed to adhere overnight. Cells were treated with either compound or DMSO for 2.5 hours at 37 °C. Puromycin was added at 1 µg/ml final concentration for the final 30 minutes of incubation. Post puromycin addition, medium was removed and cells were washed with PBS. Cells were fixed with 4% paraformaldehyde for 10 minutes. After fixation, cells were blocked with 10% goat serum in PBS with 0.4% TritonX for 1 hour at room temperature. An anti-puromycin mouse monoclonal antibody was diluted in 10% goat serum in PBS with 0.4% TritonX and cells were incubated at 4 °C overnight. Cells were washed and incubated with a goat anti-mouse AF647 secondary antibody for 1 hour at 37 °C. Post incubation cells were washed with PBS with 0.4% TritonX. Cells were incubated for 10 minutes with DAPI in PBS. Cells were then washed with PBS and imaged on a Molecular Device ImageXpress HT.ai microscope. Analysis was run in INCarta.

Non-enzymatic Glutathione Conjugation

A 1 mM DMSO Ligustrone A (**1**) solution was diluted into PBS to a final concentration of 10 µM, with and without 1 mM glutathione (Thermo Scientific, Cat# 78259) in a 100 µL reaction. The reaction was incubated at 22 °C for 1 hour before analysis via LCMS.

Aspergillus Nidulans conversion of Ligustrone A (1) to B (2)

A. nidulans cultures were performed as noted *vide supra*. At the start of the fermentation, either vehicle (DMSO) or 5 µM **1** was added to LMM with and without *A. nidulans* spores. Cultures were incubated, lyophilized, methanol extracted, and filtered through a 0.45 µm filter and analyzed via LCMS as noted *vide supra*.

P. paradoxum ligJ Overexpression RNAseq

P. paradoxum cultures were performed, as above, and fungal tissue was frozen, lyophilized, and pulverized into a powder before RNA was extracted using the TRIzol (Thermo Scientific Cat# 15596026) per manufacturer protocol. Three biological replicates were prepared per condition. Polyadenylated RNA was enriched and libraries were prepared using the NEBNext Ultra II RNA Library Prep Kit. Sequencing was performed on an Illumina NovaSeq 6000 platform to generate 2 × 150 bp paired-end reads, yielding approximately 50 million reads per sample. Raw reads were quality-filtered and adapter-trimmed using Trimmomatic.[12] Reads were aligned to a custom draft genome assembly with STAR.[13] Gene expression quantification was performed using HTSeq[14], and differential expression analysis was conducted using DESeq2.[15]

Supporting Tables

Table S1: Primers used in this study

Primer	Sequence
DY065 1031T_R-pyroA-F	gccagtcaaaataaacagtaaccagggacatcagatgctggattactaag
DY066 1029P_F-pyroA-R	acgttaccgtcacctggacctcgttgcgagtgtctacataatgaaggac
DY067 1028P_R-1028T-F	aaggattgcccgtctggaagacaagggccgcctagagcgcgtg
DY076 1036P_R	ggtgccttgtggatggggattatg
DY079 1036T R	ggtggatagccgtatctccctc
DY081 1035P R	agtactgctttcaaaagtatatcatctgctgc
DY082 1034P F	tgcgggagggtaggagggtag
DY099 1028t sceI R	attaccctgttatccctagtctgtactttgatatataatgggattttagctttggttg
DY106 1036t check F	ggaaagccgctaacactgtctatcg
DY107 1036t check F2	agttgatcctgaagcggaggacag
DY109 1036t check R2	agccagaaagggcaattctaacatactctag

Primer	Sequence
DY065 1031T_R-pyroA-F	gccagtcaaaataaacagtaaccagggacatcagatgctggattactaag
DY125 1028t check F	agtctagggtctacggttgttccac
DY130 1036t F	gctgcatcggtcatgttgttcttc
DY131 1036t R	ggtggatagccgtatctccctccag
DY134 1035p R	agtactgctttcaaaagtatatcatctgctg
DY137 1034p F	tgcgggagggtaggagggtag
DY140 1033p F	cctgtttagagtggccagaaggtg
DY140 1035P F	cctggtgtgattgggctgattagg
DY141 1033p R	tatgcaactgggccggagaattgc
DY144 1031p F	attcagcctattgagattacag
DY148 1031t F	ggcatcgtctacaagcagatgctag
DY150 1031t R	ctggttactgtttattttgactggctaatg
DY152 1029p F	aacgaggtccaggtgacggtaac
DY153 1028P R	cttgtcttccagacgggcaatcc
DY158 1034P R	tataaccacttgcctgaggatcgctc
DY168 alcAp mid R	gtggctctccgtgcggac

Primer	Sequence
DY065 1031T_R-pyroA-F	gccagtcaaaataaacagtaaccagggacatcagatgctggattactaag
DY169 1036p check F	tgcgataccttagagaaacaaatcaacatcc
DY170 1034p check R	tacatcttgtctatctactacagacacccagtc
DY171 1034p check F	gcaagctactatagatgctacatagccagatag
DY251 1036p HX0790 PKS F	gacctaaacctccataatccccatccacaaggcaccATGGCATCTCCTTGCCAACTCCTC
DY252 HX0790 R2	TCTGGGGCTTGTGGAATTGCAC
DY253 HX0790 PKS F2	TTCAGGAAAAATGCACCCGTGATACAC
DY254 HX0790 PKS 1036t R	tctatagaagaacaacatgaccgatgcagcCTATCCCATAAGAGCATGCTTCATGATCTC
DY255 1036t HX0790 FSH F	tcataatggccggcctggagggagatacggctatccaccTCAATGCGACAAAGAGGCCCG
DY256 HX0790 FSH 1035p R	ccggcctgtcctaatcagcccaatcacaccaggATGTCTCTCCCTCGCATAGCATGTTTC
DY257 1035p HX790 RPL10 F	cgtggctaggcagcagatgatatacttttgaaagcagtactTCATACGCTCGCCTCCACG
DY258 HX0790 RPL10 1034p R	tatcaactacctagctaccctcctaccctcctaccctcccgcaATGGCGCGCAGACCTGC
DY259 1031t HX0790 EthD F	tccattagccagtcaaaataaacagtaaccagCTAAGCACTGGCAAATGTGCCATTAGAG
DY260 HX0790 EthD	actgcactgaaccacgttaccgtcacctggacctcgttATGGTCACCGCTACT

Primer	Sequence
DY065 1031T_R-pyroA-F	gccagtcaaaataaacagtaaccagggacatcagatgctggattactaag
1029p R	CCCACTC
DY261 1034p HX0790 MT1 F	tgattatagagcgatcctcaggcaagtggttataTCAGACCACCTCGATCTCCAGAATTG
DY262 HX0790 MT1 1033p R	gcagataacacacacaccttctggccactctaaacaggATGGGCCCATTGAACAAGGGTG
DY263 1033p HX0790 P450 F	tgatttgcaattctccggcccagttgcataATGTTTCCCACTCTAGTATCATGTGTTCTC
DY264 HX0790 P450 1031p R	ggattacttccgtggctgtaatctcaataggctgaatTCAAGCCCGACGATGTTTCAACC
DY265 1031p HX0790 MT2 F	gcacttgaagtcttgtctacatcccgaatccaggcgcaATGGGCCCATTGAACAAGGGTG
DY266 HX0790 MT2 1031t R	aatgtgtgcctagcatctgcttgtagacgatgccTCAGACCACCTCGATCTCCAGAATTG
DY289 str4 check F	CGTAGTCCTGTCGAGCCACTGTC
DY290 str4 check R	GCTGAAACCGCAGTCGAGTCC
DY304 1036t mid check R	cccctccgcttttagcctgtg
DY364 1033p check F	aatacggccttgatcagccaatcac
DY368 hx790 rpl10 check F	GCATGGGAAGTCATCCACAGAAGC
DY369 hx0790 MT1 check R	GCCATGGTGCAAACTAGACAGATGATTC

Primer	Sequence
DY065 1031T_R-pyroA-F	gccagtcaaaataaacagtaaccagggacatcagatgctggattactaag
DY373 1036t check R	gcgaacaaactggtgagtttctgtcc
DY385 1028p hx0790 MT2 F	tctgacagaaggattgcccgtctggaagacaagATGGGATCCATCAGTGATTTTTCGTCG
DY387 hx0790 MT2 term pyroA R	tgaccttacattaccttagtaatccagcatctgatgtccATGGCAGCCGCTATGCCCTTC
DY388 hx0790 MT2 F2	GACATCGATGGATTTGCTCATGATGACAG
DY389 HX0790 MT2 R2	TGACTTCATCAGCCAACTCAGCCTC
DY432 1034p F2	GATTATAGAGCGATCCTCAGGCAAGTGG
DY459 coxAp str4 TF F	CTCCACGCCTTGTCCGTTGGCATTGCACCCACAATGTCGATGTCTCCCTTGAGAGATCAC
DY461 str4 TF tAN0717 R2	gatgtatcctaagtcggtaacctcattctcggcacCTATTTCGGCGCCTGCGTACAGACC
DY538 1036p F	AATGACTGGTCCGTCCGTACTTAGAAAGG

Table S2: NMR spectroscopic data of ligustrone A (**1**)

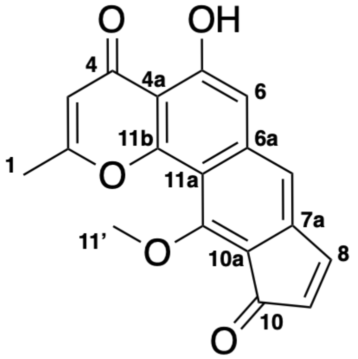

1

	Ligustrone A ($CDCl_3$)[16]		Euplectin ($CDCl_3$)[17]		Found (CD_3CN)	
Position	δ_C	δ_H, m(*J* in Hz)	δ_C	δ_H, m(*J* in Hz)	δ_C	δ_H, m(*J* in Hz)
1	-	2.50	20.6	2.53 (s, 3H)	20.14	2.55 (s, 3H)
2	-	-	168.0	-	169.17	-
3	-	6.28	110.3	6.27 (s, 3H)	110.1	6.58 (s, 1H)
4	-	-	182.7	-	182.72	-
4a	-	-	110.2	-	111.73	-
5	-	-	160.3	-	159.11	-
6	-	6.86	110.1	6.84 (s, 1H)	108.91	7.17 (s, 1H)
6a	-	-	144.1	-	144.82	-
7	-	6.94	115.6	6.87 (s, 1H)	117.69	7.37 (s, 1H)
7a	-	-	142.5	-	143.43	-
8	-	7.54	147.2	7.49 (d, *J*=5.7Hz, 1H)	148.44	7.91 (d, *J*=5.7 Hz, 1H)
9	-	6.13	132.3	6.19 (d, *J*=5.7Hz, 1H)	132.65	6.25 (d, *J*=5.7 Hz, 1H)
10	-	-	198.4	-	193.28	-
10a	-	-	107.7	-	110.10	-
11	-	-	155.6	-	156.12	-
11a	-	-	108.3	-	108.91	-

	Ligustrone A ($CDCl_3$)[16]		Euplectin ($CDCl_3$)[17]		Found (CD_3CN)	
Position	δ_C	δ_H, m(*J* in Hz)	δ_C	δ_H, m(*J* in Hz)	δ_C	δ_H, m(*J* in Hz)
11b	-	-	156.8	-	156.47	-
11’	-	4.18	-*	-*	62.42	4.10 (s, 3H)
5-OH	-	13.08	-	13.09 (br s, 1H)	-	13.33 (br s, 1H)

*^{13}C NMR data not reported

**Euplectin is the de-methylated form of ligustrone A, reported for similarity comparison

Table S3: NMR spectroscopic data of ligustrone B (**2**)

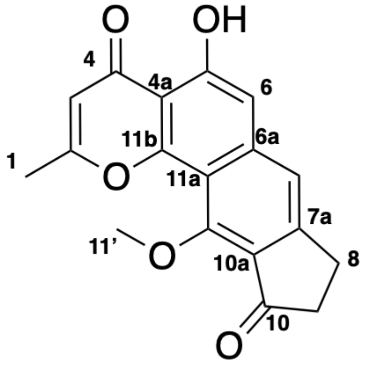

2

	Literature ($CDCl_3$)[16]		Coneuplectin ($CDCl_3$)[17]		Found (CD_3CN)	
Position	δ_C	δ_H, m(*J* in Hz)	δ_C	δ_H, m(*J* in Hz)	δ_C	δ_H, m(*J* in Hz)
1	-	2.55	20.6	2.56 (s, 3H)	20.67	2.56 (s, 3H)
2	-	-	167.5	-	169.76	-
3	-	6.36	110.9	6.31 (s, 1H)	111.55	6.42 (s, 1 H)
4	-	-	182.4	-	184.08	-
4a	-	-	109.8	-	111.47	-
5	-	-	159.0	-	159.18	-
6	-	6.90	106.5	6.82 (s, 1H)	106.44	6.96 (s, 1H)
6a	-	-	144.6	-	144.44	-
7	-	7.35	113.9	7.05 (s, 1H)	119.57	7.48 (s, 1H)

	Literature ($CDCl_3$)[16]		Coneuplectin ($CDCl_3$)[17]		Found (CD_3CN)	
Position	δ_C	δ_H, m(*J* in Hz)	δ_C	δ_H, m(*J* in Hz)	δ_C	δ_H, m(*J* in Hz)
7a	-	-	152.0	-	155.83	-
8	-	3.17	25.4	3.16-3.20 (m, 2H)	25.85	3.22-3.16 (m, 2H)
9	-	2.76	36.1	2.78-2.82 (m, 2H)	37.97	2.70-2.65 (m, 2H)
10	-	-	209.6	-	204.09	-
10a	-	-	116.4	-	125.42	-
11	-	-	157.5	-	158.40	-
11a	-	-	106.2	-	112.46	-
11b	-	-	158.7	-	158.74	-
11’	-	4.14	-	-	63.18	4.10 (s, 3H)
5-OH	-	12.90	-	12.93 (br s, (1H)	-	13.09 (s, 1H)

*^{13}C NMR data not reported

**Coneuplectin is the de-methylated form of ligustrone B, reported for similarity comparison

Table S4: NMR spectroscopic data of topopyrone C (**4**)

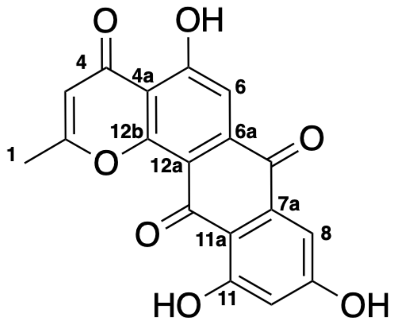

4

	Literature ($CDCl_3$)[18]		Found (Acetone-*d6*)	
Position	δ_C	δ_H, m(*J* in Hz)	δ_C	δ_H, m(*J* in Hz)
1	20.122	2.494 (s)	20.71	2.59 (s, 3H)
2	166.66	-	171.50	-
3	112.52	6.189 (s)	110.84	6.47 (s, 1H)
4	175.65	-	185.51	-
4a	121.18	-	113.51	-
5	159.48*	-	*	-
6	116.94	7.835 (s)	110.59	7.45 (s, 1H)
6a	136.85	-	135.10	-
7	180.31	-	182.31	-
7a	134.70	-	135.10	-
8	118.33	7.957 (d, 2.44)	108.81	7.21 (s, 1H)
9	154.70*	-	165.78	-
10	123.94	7.306 (d, 2.44)	109.79	6.69 (s, 1H)
11	151.36*	-	166.39	-
11a	123.80	-	110.83	-
12	178.29	-	179.16	-
12a	121.12	-	*	-
12b	156.58	-	*	-
5-OH	-	-	-	13.32 (s, 1H)

	Literature ($CDCl_3$)[18]		Found (Acetone-*d6*)	
Position	δ_C	δ_H, m(*J* in Hz)	δ_C	δ_H, m(*J* in Hz)
9-OH	-	-	-	-
11-OH	-	-	-	-

*Poor solubility of topopyrone C. Acetylated derivative reported

Table S5: NMR spectroscopic data of **6**

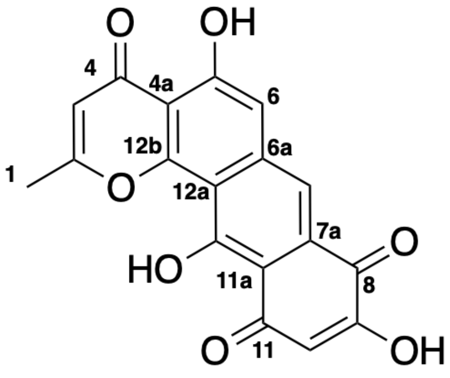

6

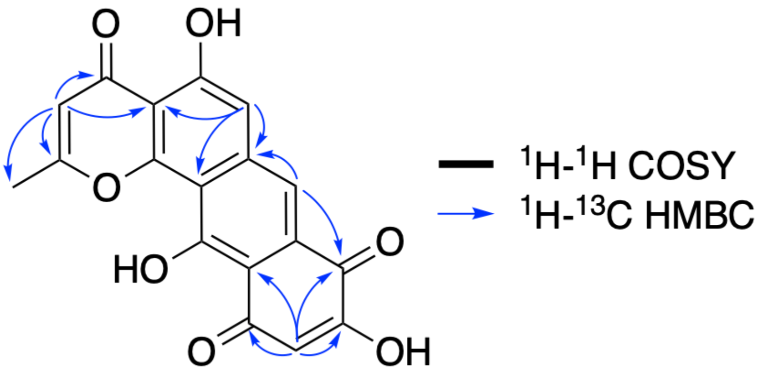

	Found (DMSO-*d6*)	
Position	δ_C	δ_H, m(*J* in Hz)
1	20.24	2.54 (s, 3H)
2	168.86	-
3	110.31	6.57 (s, 1H)
4	182.51	-
4a	110.37	-
5	157.28	-
6	108.35	7.21 (s, 1H)
6a	139.45	-
7	116.00	7.64 (s, 1H)
7a	131.85	-
8	185.79	-
9	162.85	-

	Found (DMSO-*d6*)	
Position	δ_C	δ_H, m(*J* in Hz)
10	105.18	5.32 (s, 1H)
11	184.85	-
11a	108.16	-
12	174.21	-
12a	111.58	-
12b	156.27	-
5-OH	-	13.2 (s, 1H)
9-OH	-	-
12-OH	-	-

Table S6: NMR spectroscopic data of **8**

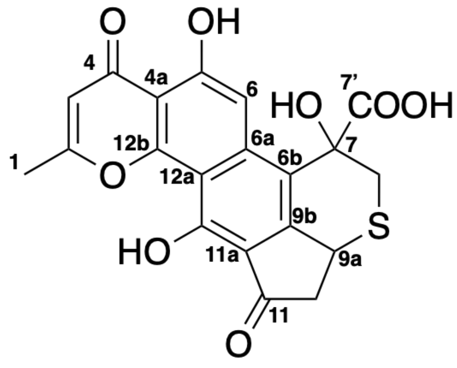

8

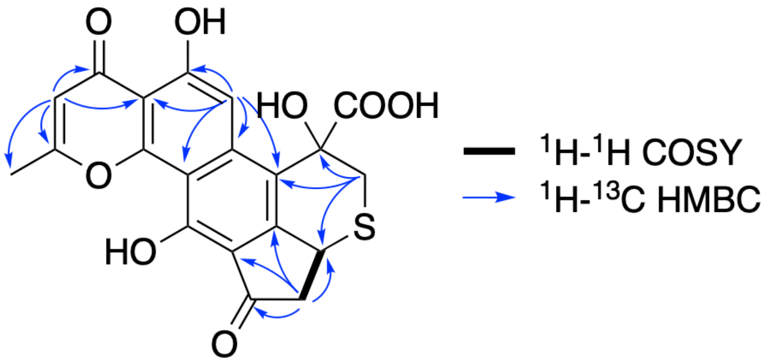

	Found (CD_3CN)	
Position	δ_C	δ_H, m(*J* in Hz)
1	20.71	2.50 (s, 3H)
2	169.97	-
3	111.52	6.38 (s, 1H)
4	183.51	-
4a	110.53	-
5	160.79	-

	Found (CD_3CN)	
Position	$δ_C$	$δ_H$, m(*J* in Hz)
6	104.80	7.09 (s, 1H)
6a	143.65	-
6b	120.75	-
7	71.98	-
8	44.59	3.66 (d, *J*=14.2 Hz, 1H); 3.26 (d, *J*=14.2 Hz, 1H)
9	-	-
9a	40.33	4.47 (t, *J*= 7.4 Hz, 1H)
9b	151.37	3.13 (dd, *J*=18.0, 7.4 Hz, 1H), 2.67 (dd, *J*=18.0, 7.4 Hz, 1H)
11	205.72	-
11a	114.28	-
12	158.93	-
12a	108.44	-
12b	158.71	-
7’	176.95	-
5-OH	-	13.11 (s, 1H)
12-OH	-	-

Table S7: NMR spectroscopic data of **9**

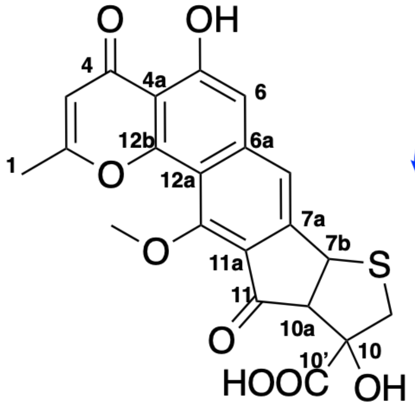

9

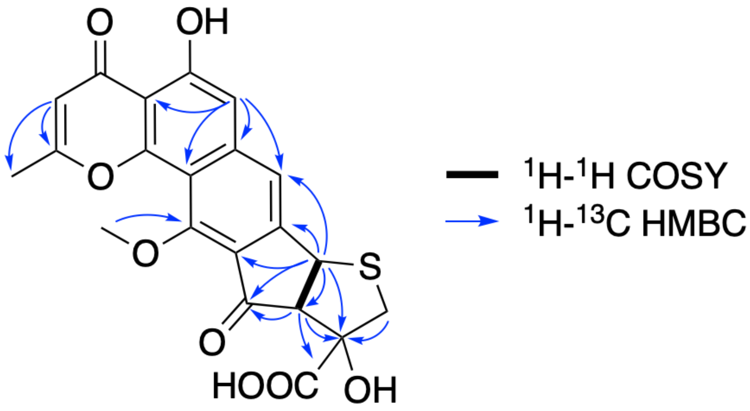

	Found (CD_3CN)	
Position	δ_C	δ_H, m(*J* in Hz)
1	20.68	2.55 (s, 3H)
2	169.93	-
3	111.90	6.42 (s, 1H)
4	184.03	-
4a	111.62	-
5	159.84	-
6	106.85	6.97 (s, 1H)
6a	145.02	-
7	119.04	7.44 (s, 1H)
7a	155.21	-
7b	48.38	5.25 (d, *J*=8.8 Hz, 1H)
8	-	-
9	46.76	3.60 (d, *J*=8.6 Hz, 1H);<br>3.03 (d, *J*=8.6 Hz, 1H)
10	84.86	-
10a	64.04	3.88 (d, *J*=8.6 Hz, 1H)
11	198.88	-
11a	124.26	-

12	158.63	-
12a	112.93	-
12b	158.63	
10’	173.96	-
11’	63.47	4.10 (s, 3H)
5-OH	-	13.16 (s, 1H)
10-OH	-	-
10’-OH	-	-

Table S8: NMR spectroscopic data of **10**

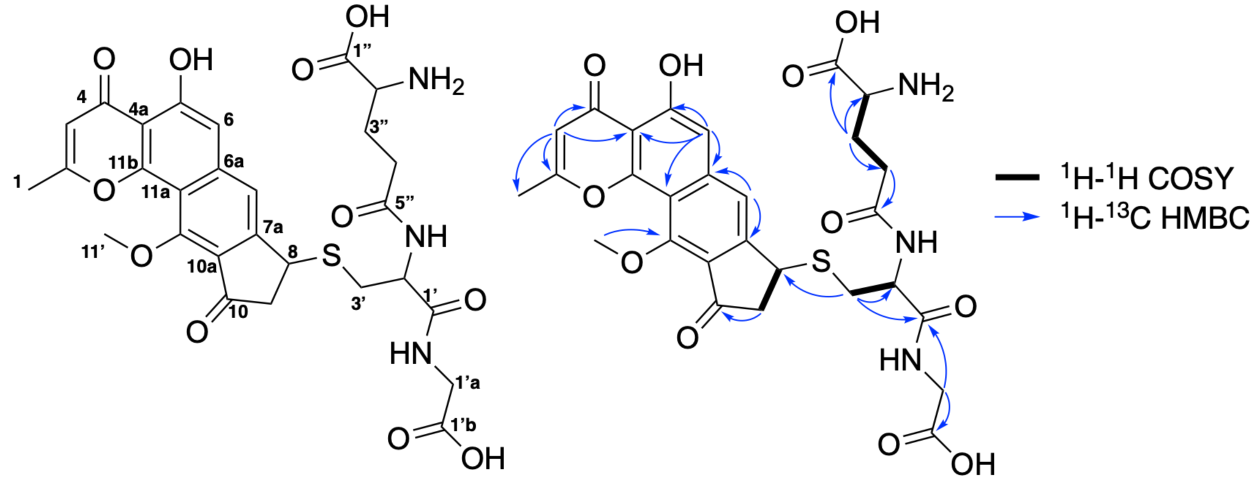

10

	Found (CD_3OD)	
Position	δ_C	δ_H, m(*J* in Hz)
1	20.49	2.62 (s, 1H)
2	170.36	-
3	111.81	6.49 (s, 1H)
4	184.40	-
4a	111.81	-

	Found (CD_3OD)	
Position	δ_C	δ_H, m(*J* in Hz)
5	159.74	-
6	107.71	7.10 (s, 1H)
6a	145.15	-
7	120.71	7.73 (s, 1H)
7a	124.45	-
8	41.61	4.79-4.74 (m, 1H)
9	48.08	3.34 (m, 1H); 2.82 (dd, *J*=18.9, 3.2 Hz, 1H)
10	202.48	-
10a	106.06	-
11	159.48	-
11a	113.27	-
11b	155.00	-
11’	63.62	-
1’	172.17	-
1’a	43.94	-
1’b	175.13	-
2’	54.10	4.70 (dd, *J*=9.3, 5.1 Hz, 1H)
3’	34.29	3.22 (dd, *J*=13.8, 6.8 Hz, 1H); 2.90 (dd, *J*=13.8, 6.8 Hz, 1H)
1”	173.91	-
2”	55.64	3.65 (t, *J*=6.1 Hz, 1H)
3”	27.93	2.15 (m, 2H)
4”	33.30	2.58 (t, *J*=7.2 Hz, 2H)
5”	175.44	-
5-OH	-	-

NMR Spectra

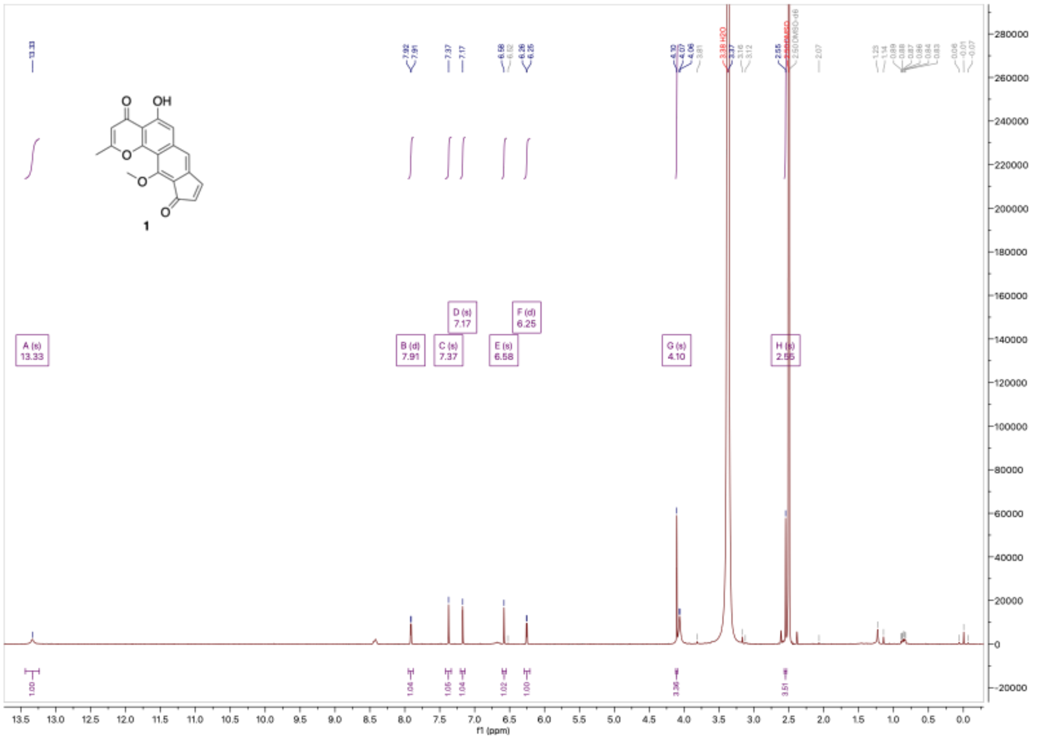

Figure S7: ^{1}H NMR Spectrum of ligustrone A **(1)** (600MHz, DMSO-*d6*)

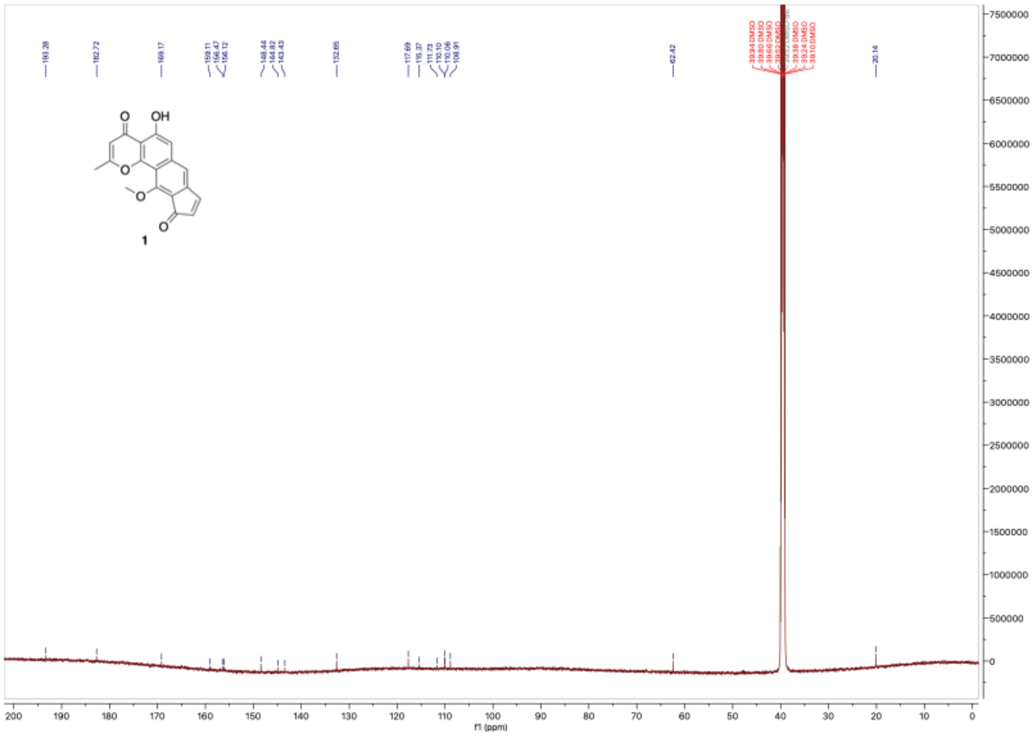

Figure S8: ^{13}C NMR Spectrum of ligustrone A **(1)** (150MHz, DMSO-*d6*)

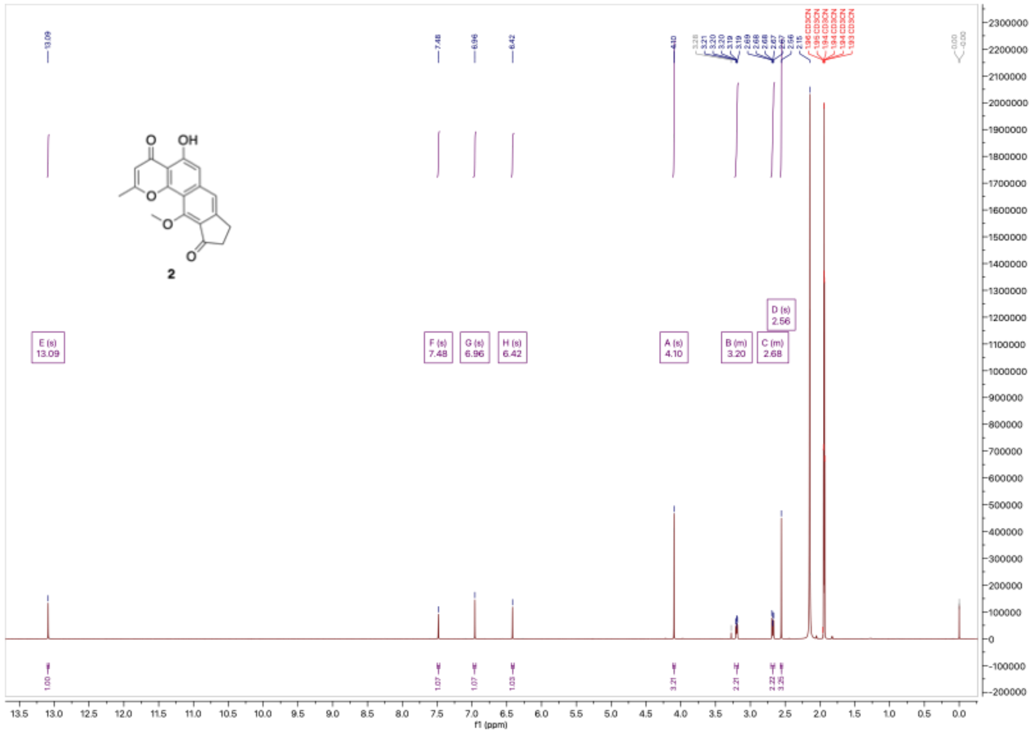

Figure S9: ^{1}H NMR Spectrum of ligustrone B **(2)** (600MHz, CD_3CN)

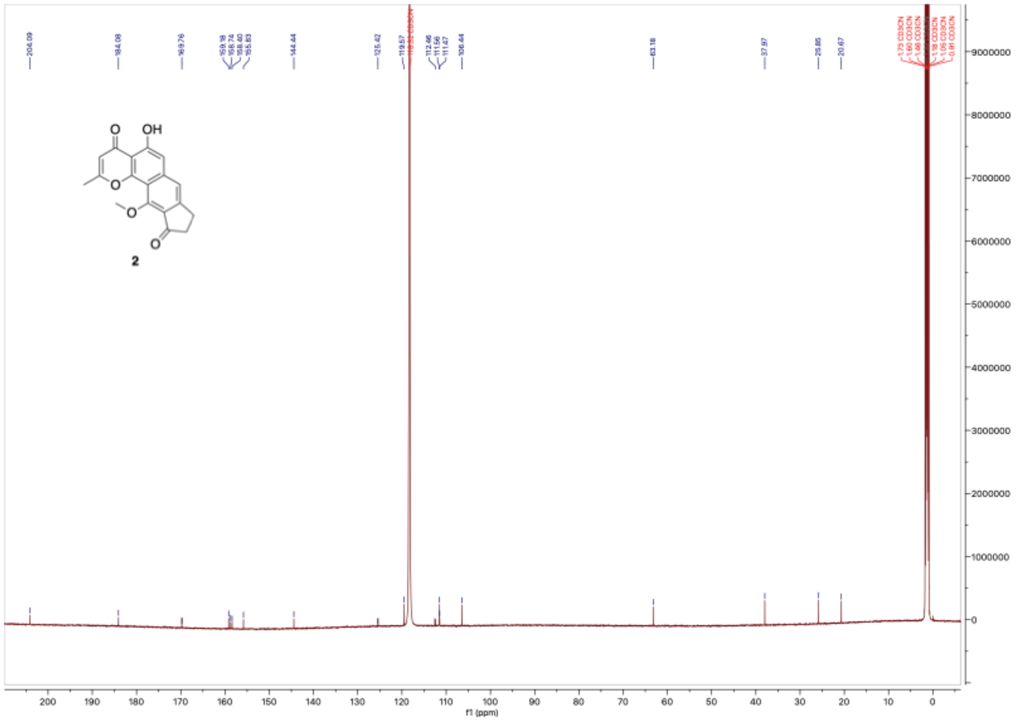

Figure S10: ^{13}C NMR Spectrum of ligustrone B (**2)** (150MHz, CD_3CN)

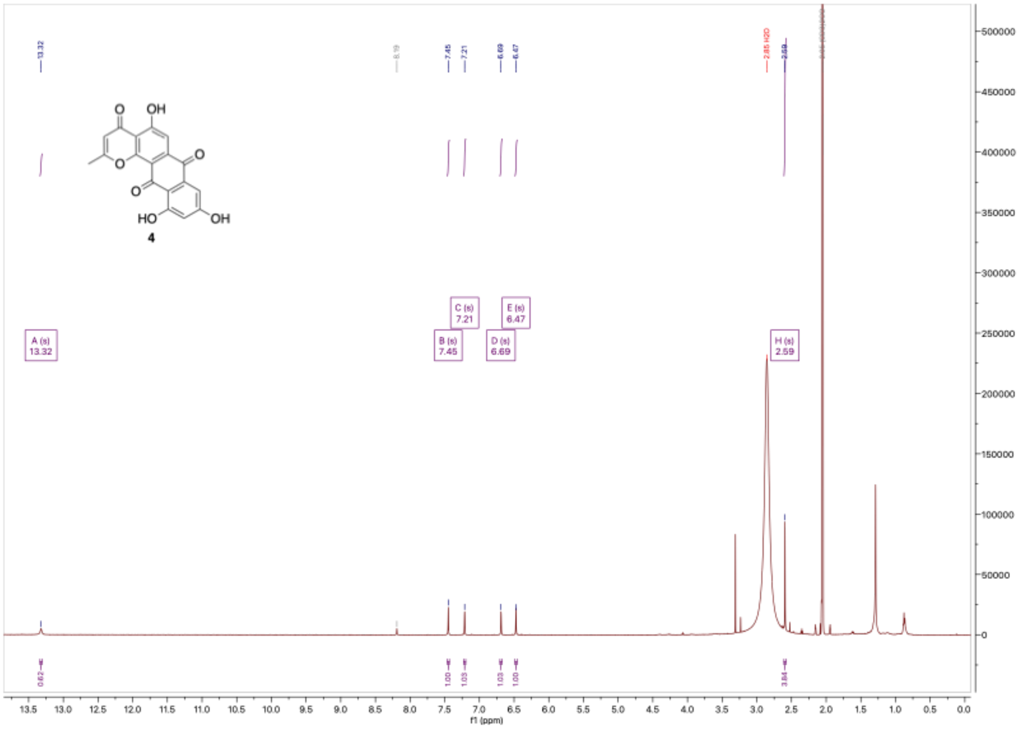

Figure S11: ^{1}H NMR Spectrum of topopyrone C (**4**) (600MHz, acetone-*d6*)

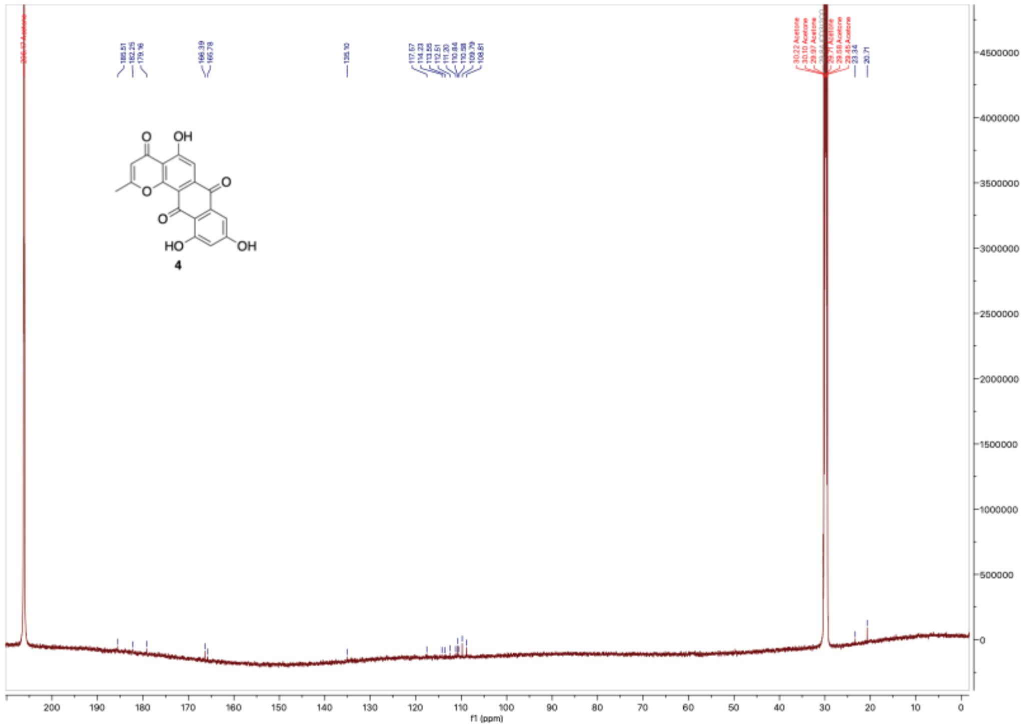

Figure S12: ^{13}C NMR Spectrum of topopyrone C (**4**) (150MHz, acetone-*d6*)

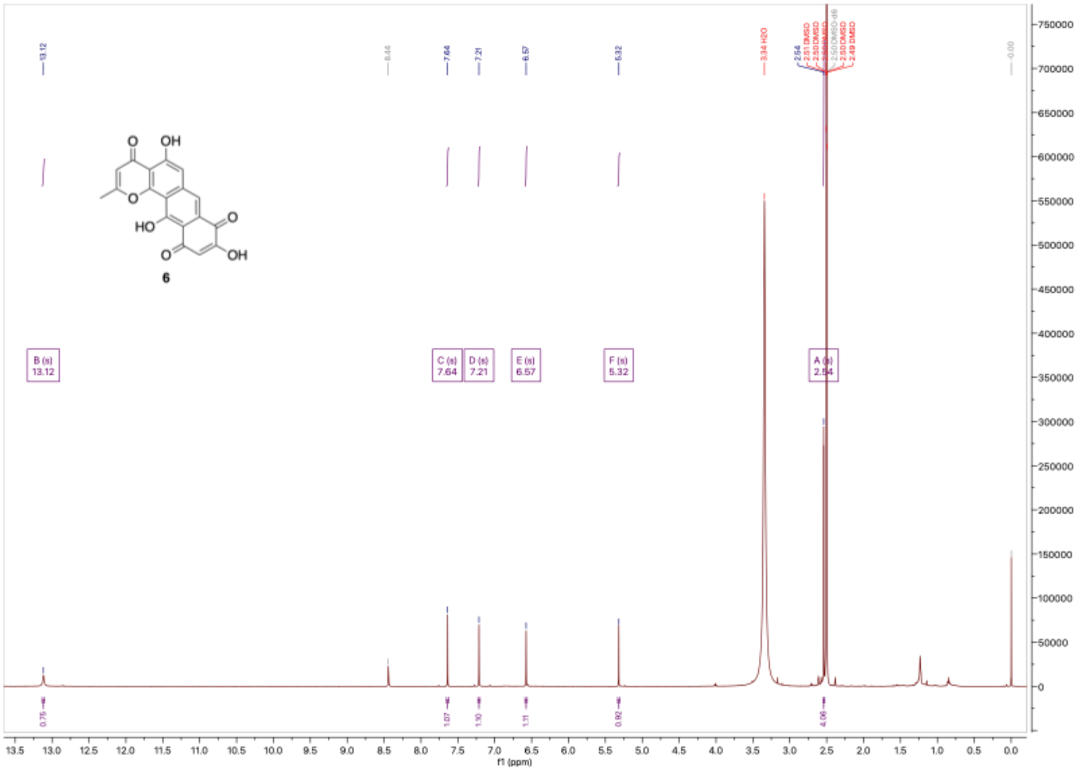

Figure S13: ^{1}H NMR Spectrum of **6** (600MHz, DMSO-*d6*)

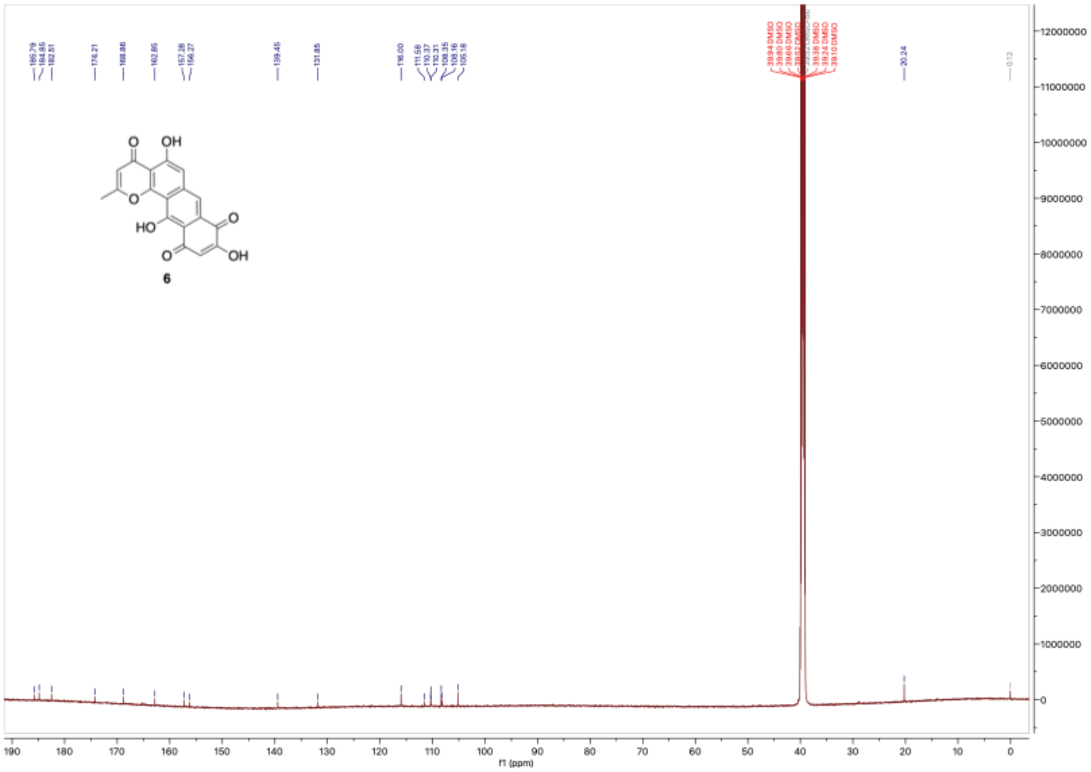

Figure S14: ^{13}C NMR Spectrum of **6** (150MHz, DMSO-*d6*)

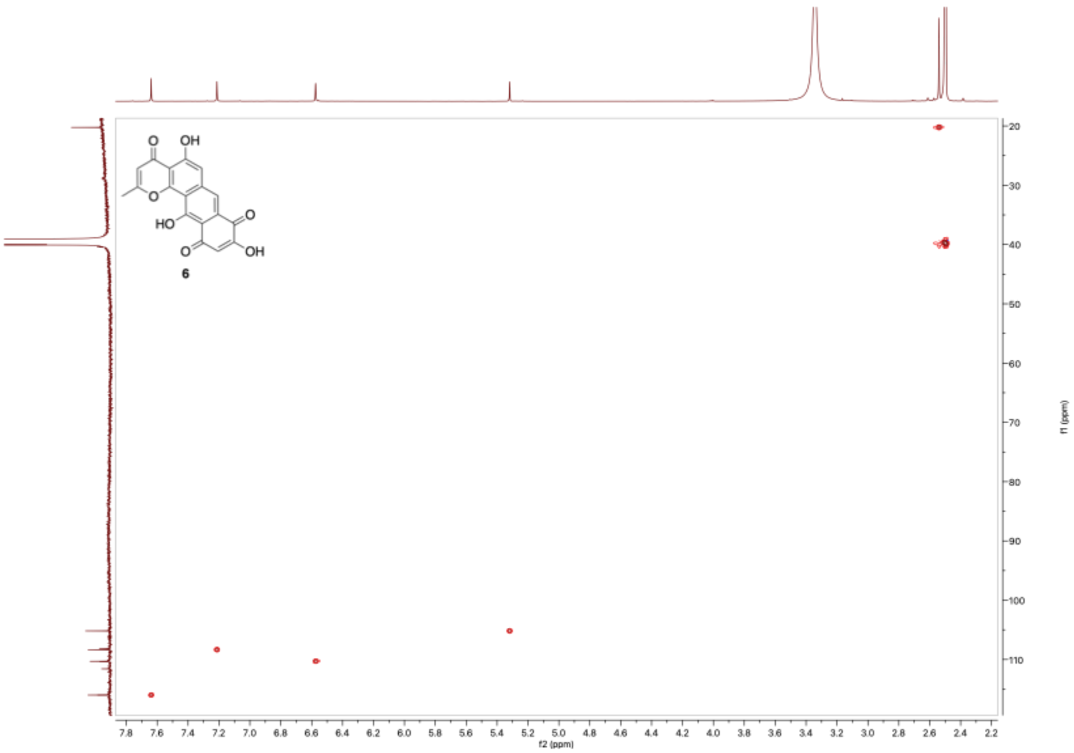

Figure S15: ^{1}H-^{13}C HSQC NMR Spectrum of **6** (600MHz, DMSO-*d6*)

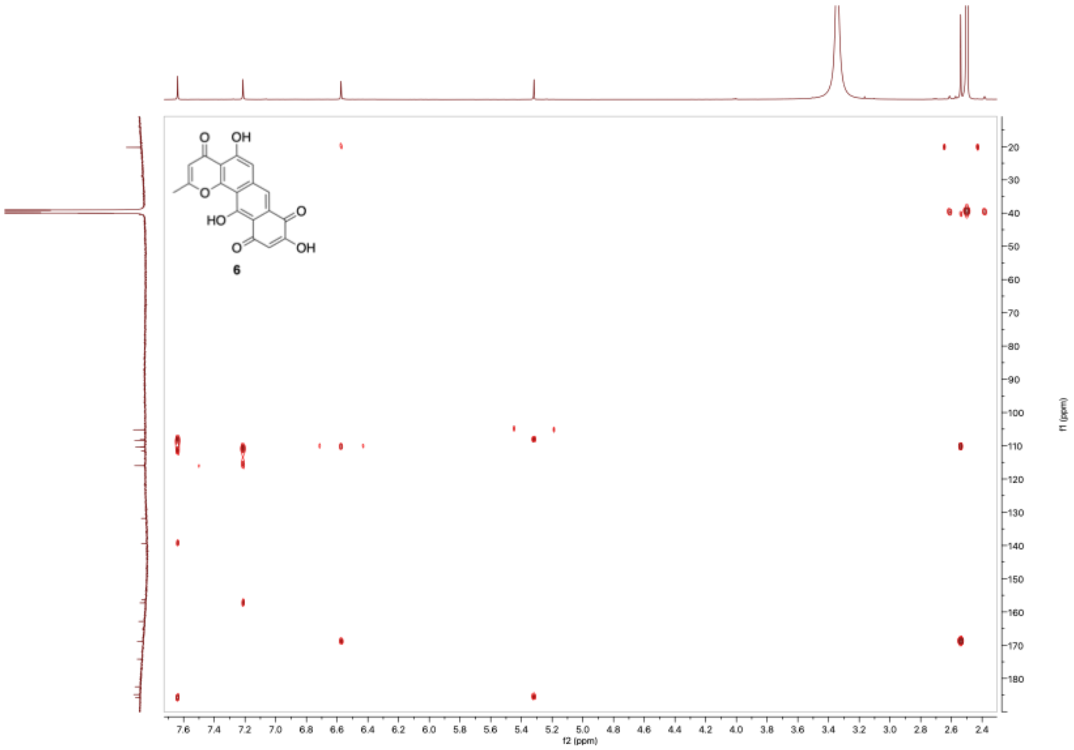

Figure S16: ^{1}H-^{13}C HMBC NMR Spectrum of **6** (600MHz, DMSO-*d6*)

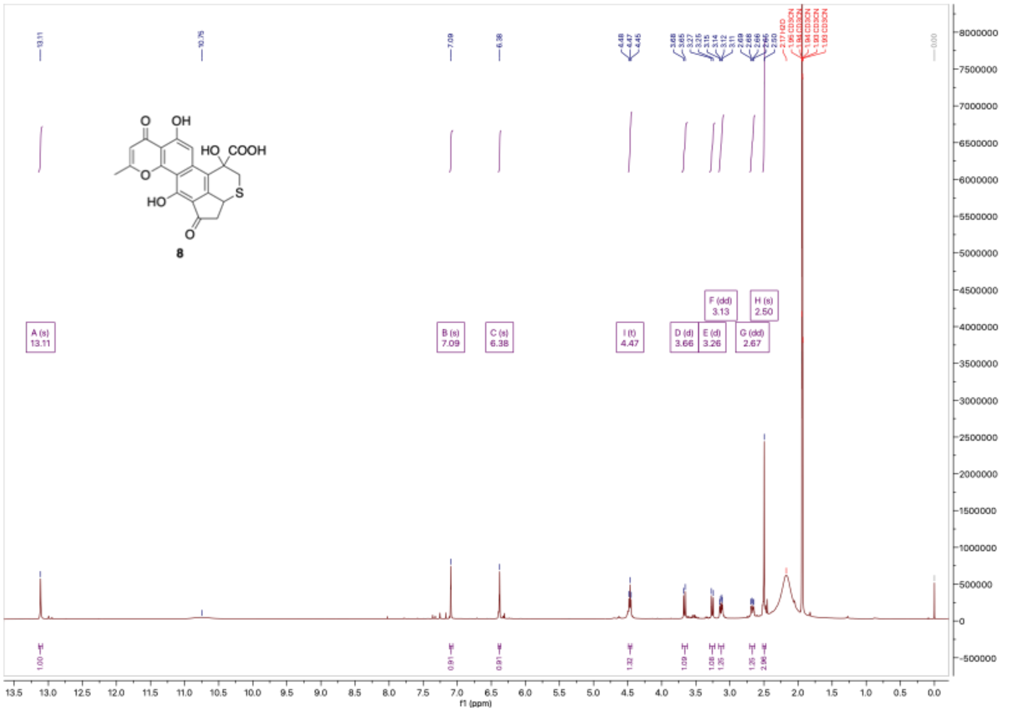

Figure S17: ^{1}H NMR Spectrum of **8** (600MHz, CD_3CN)

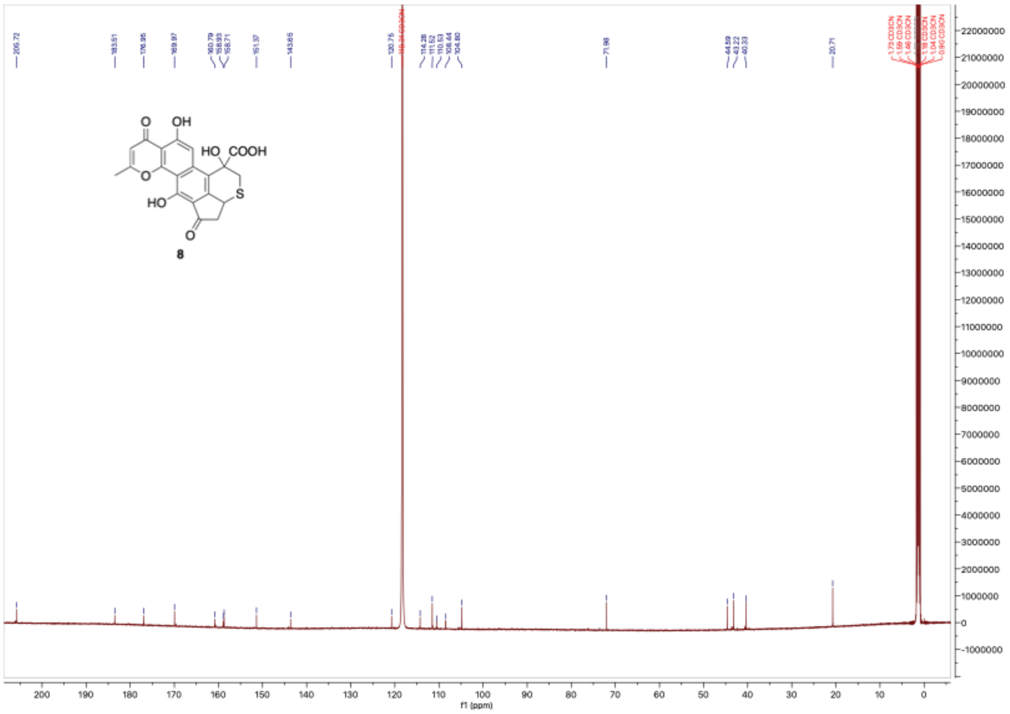

Figure S18: ^{13}C NMR Spectrum of **8** (150MHz, CD_3CN)

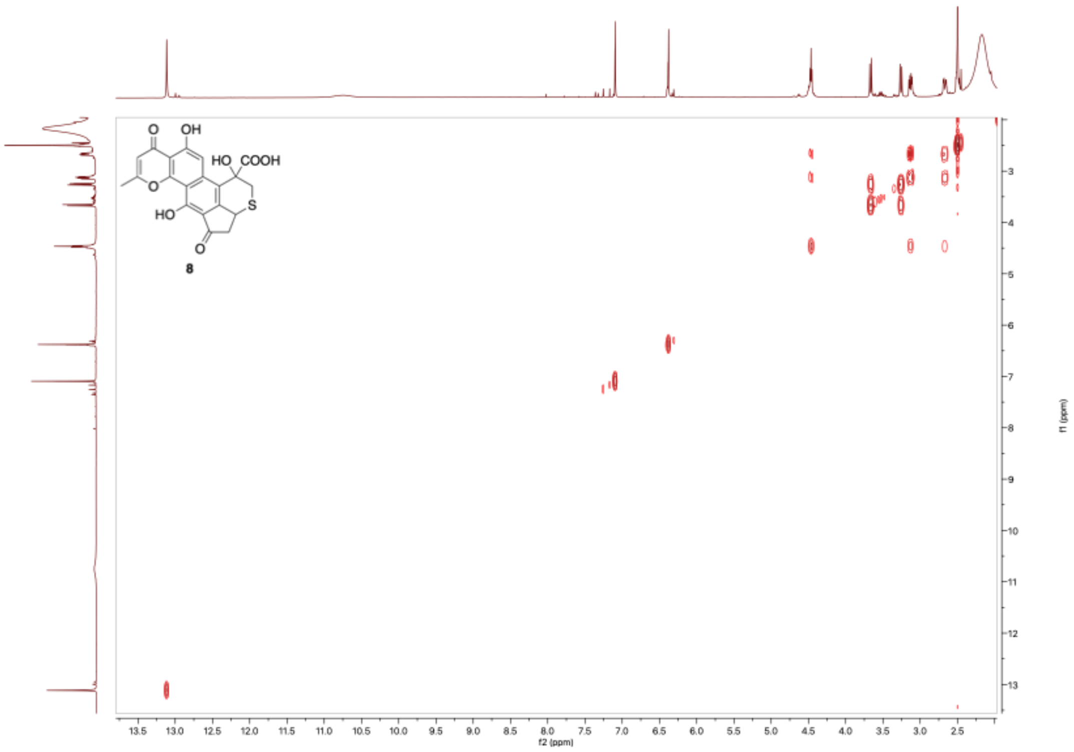

Figure S19: ^1H-^1H COSY NMR Spectrum of **8** (600MHz, CD_3CN)

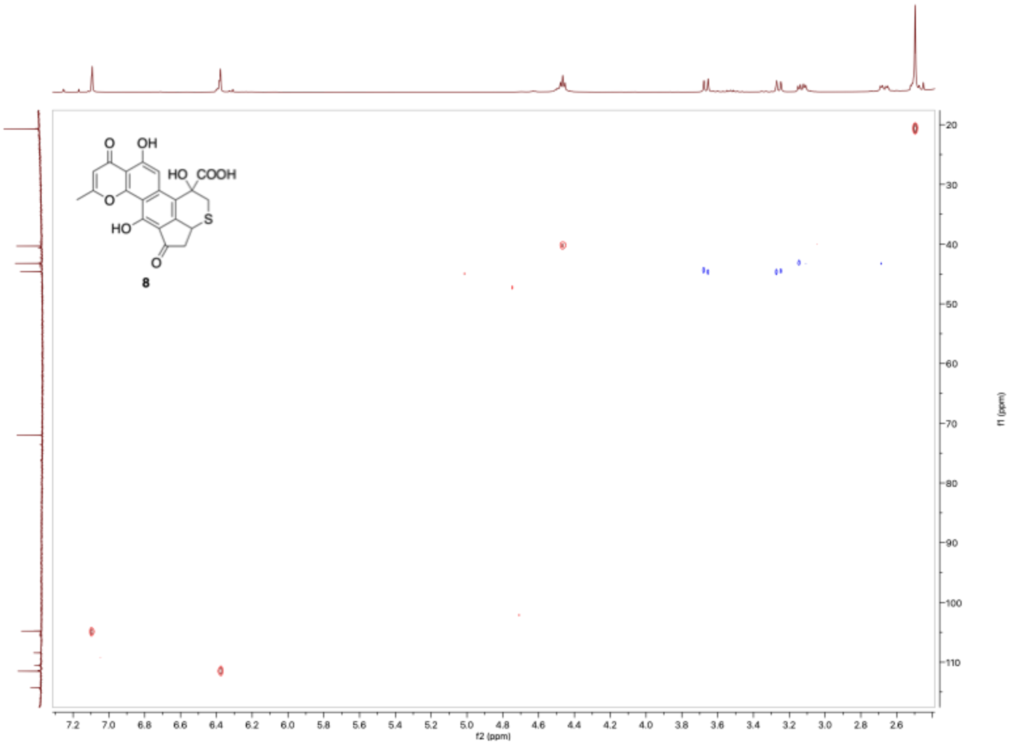

Figure S20: ^1H-^{13}C HSQC NMR Spectrum of **8** (600MHz, CD_3CN)

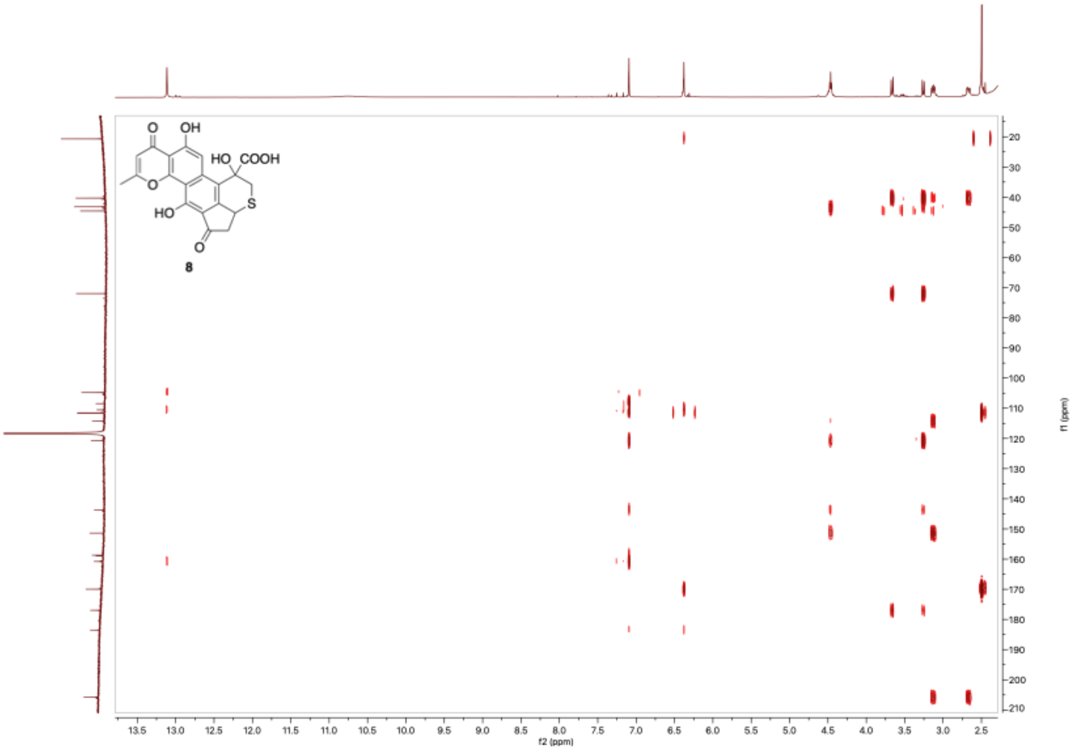

Figure S21: ^1H-^{13}C HMBC NMR Spectrum of **8** (600MHz, CD_3CN)

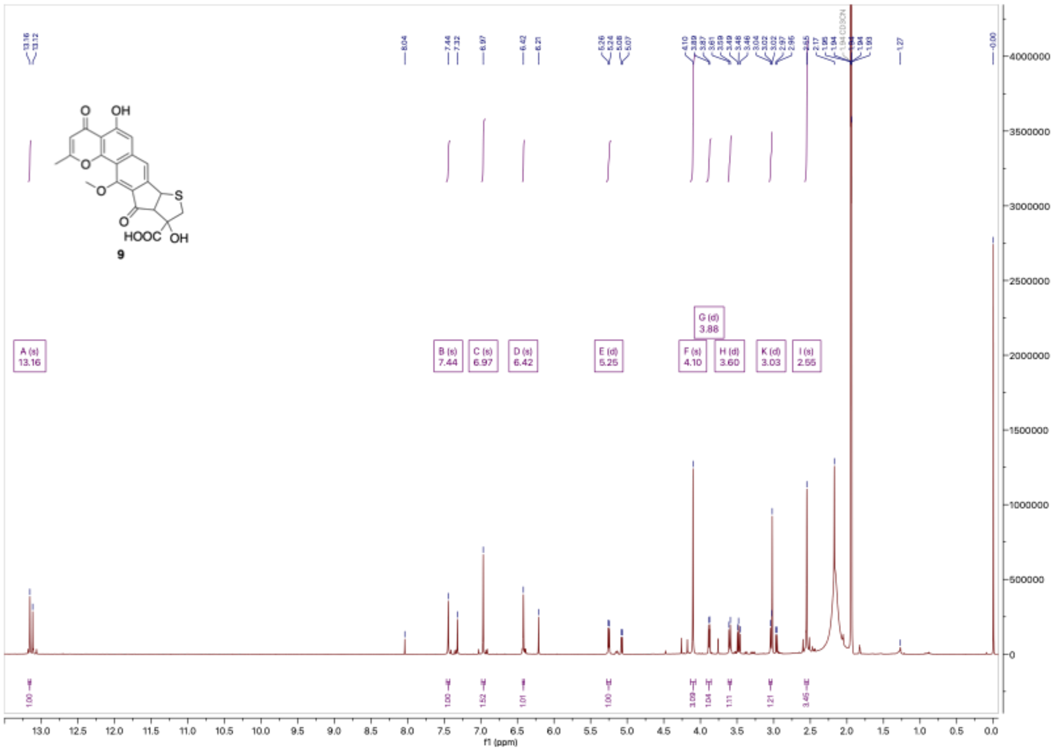

Figure S22: ^1H NMR Spectrum of **9** (600MHz, CD_3CN)

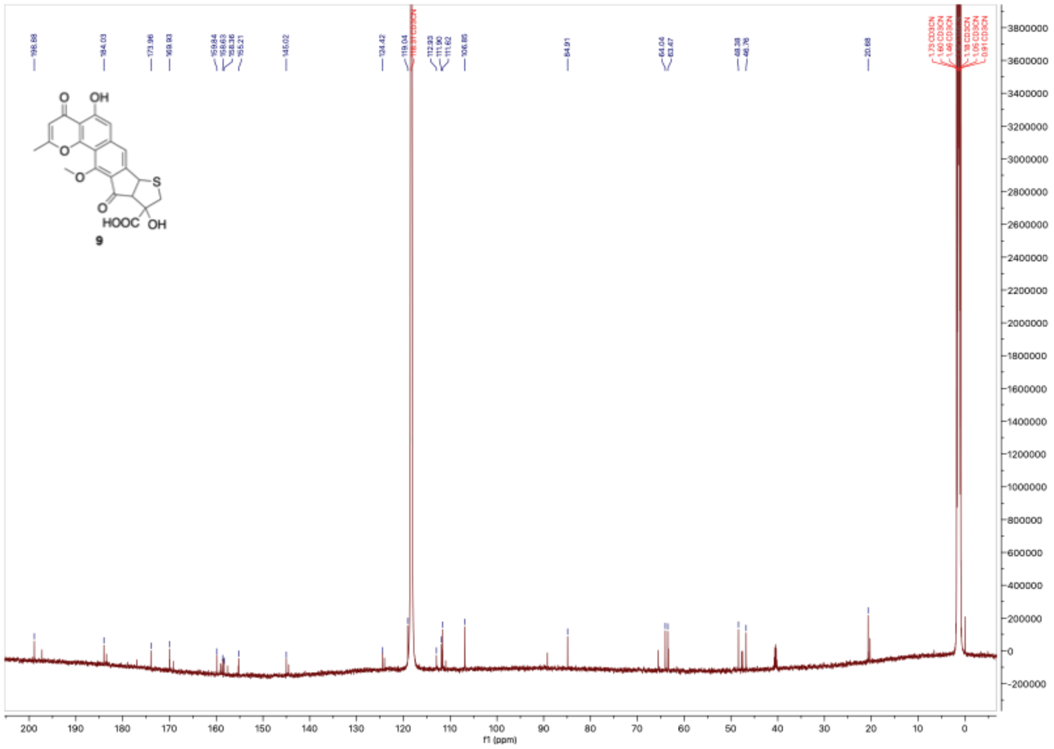

Figure S23: ^{13}C NMR Spectrum of **9** (150MHz, CD_3CN)

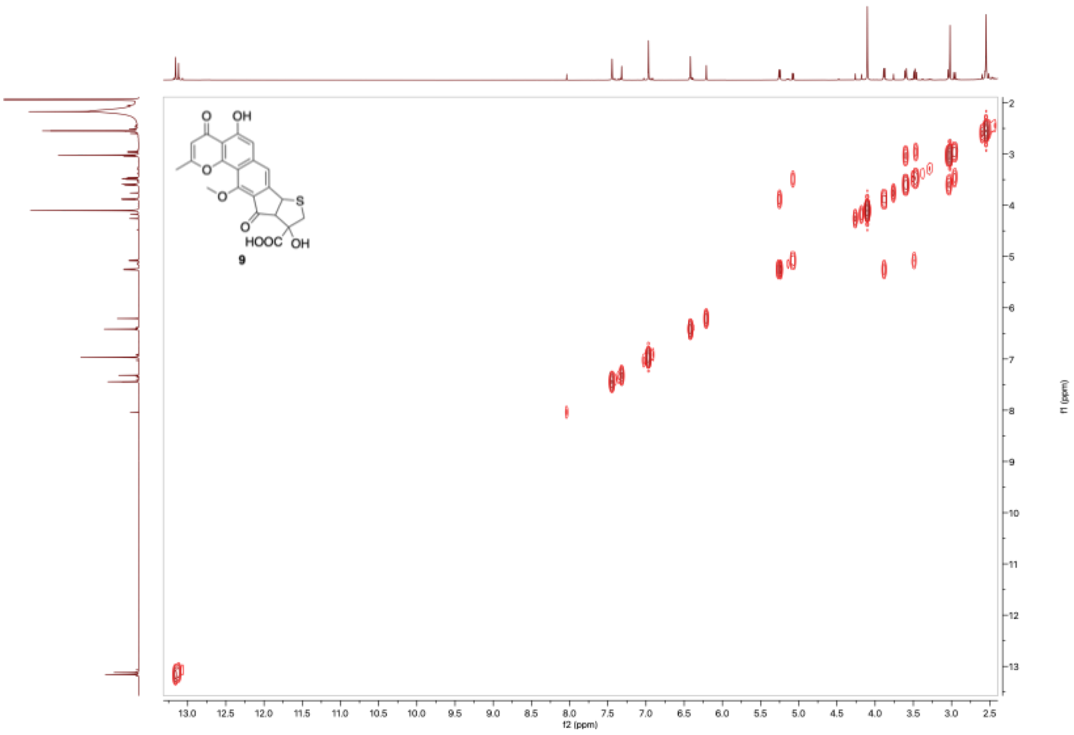

Figure S24: ^{1}H-^{1}H COSY NMR Spectrum of **9** (600MHz, CD_3CN)

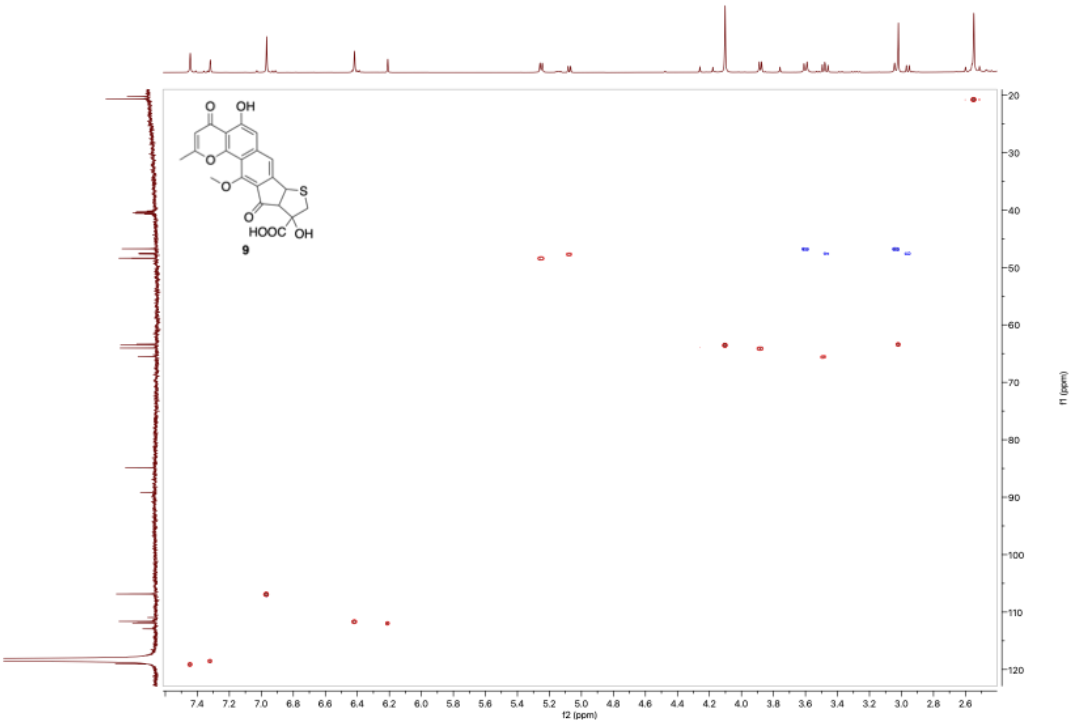

Figure S25: ^{1}H-^{13}C HSQC NMR Spectrum of **9** (600MHz, CD_3CN)

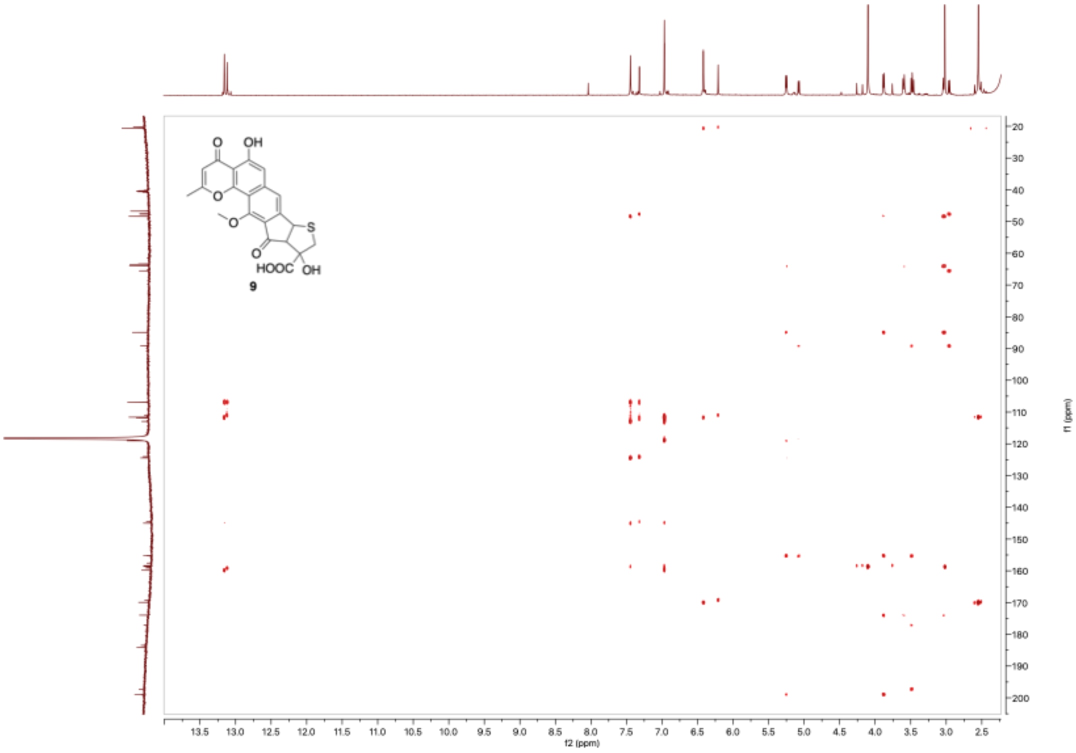

Figure S26: ^{1}H-^{13}C HMBC NMR Spectrum of **9** (600MHz, CD_3CN)

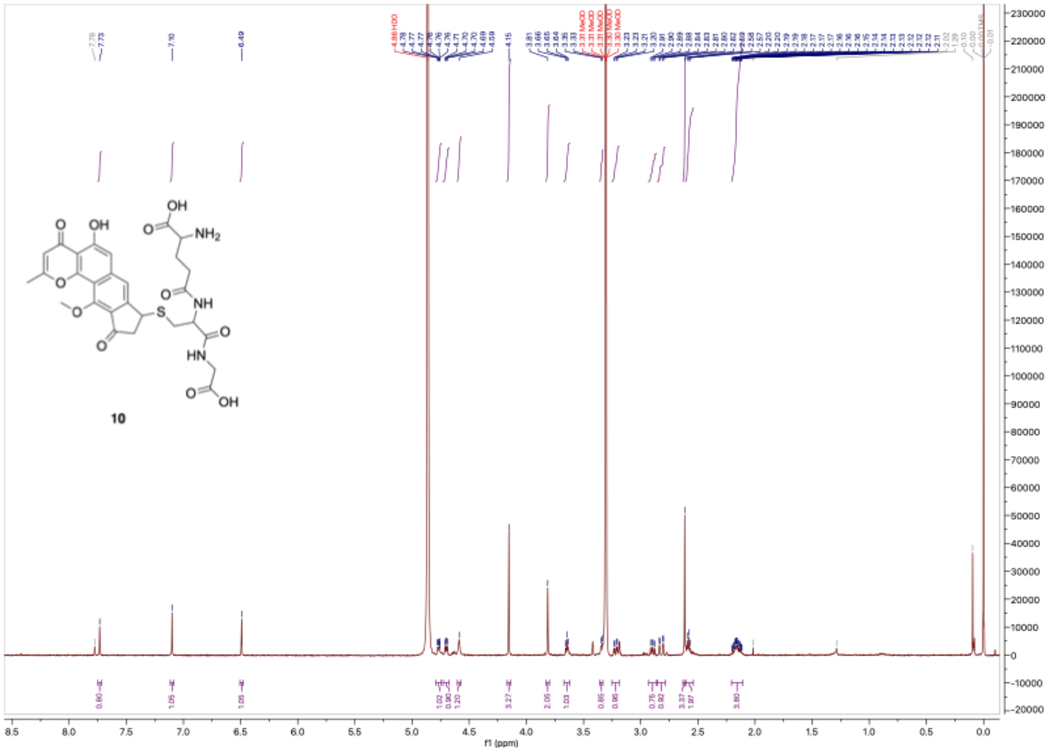

Figure S27: ^{1}H NMR Spectrum of **10** (600MHz, CD_3OD)

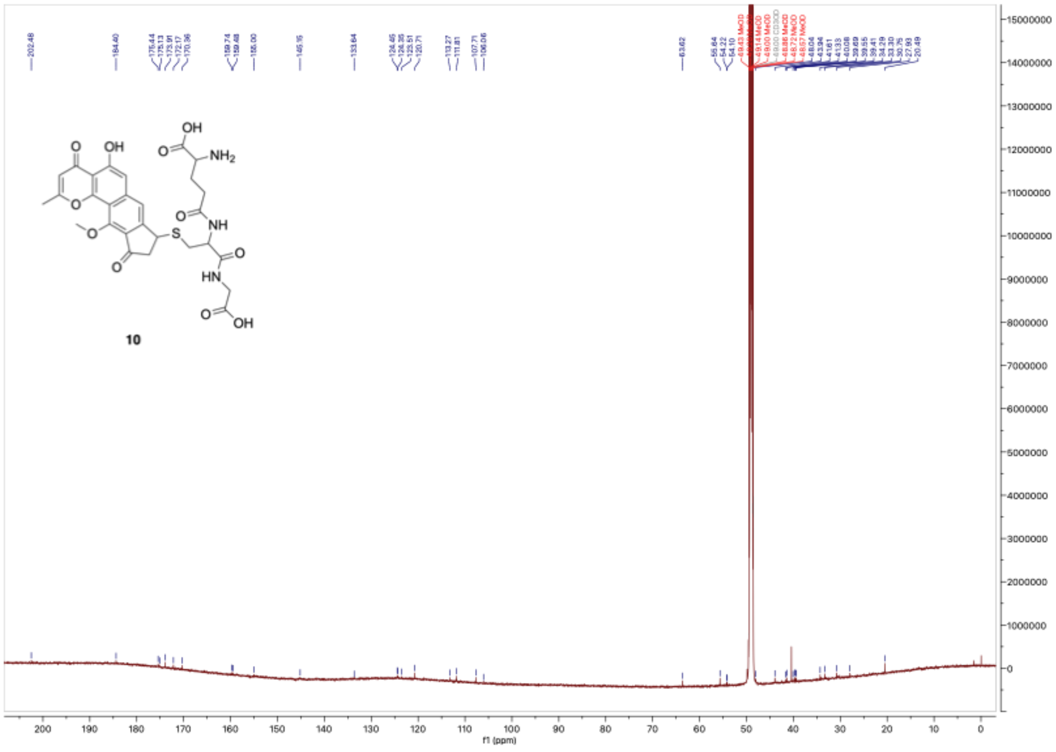

Figure S28: ^{13}C NMR Spectrum of **10** (150MHz, CD_3OD)

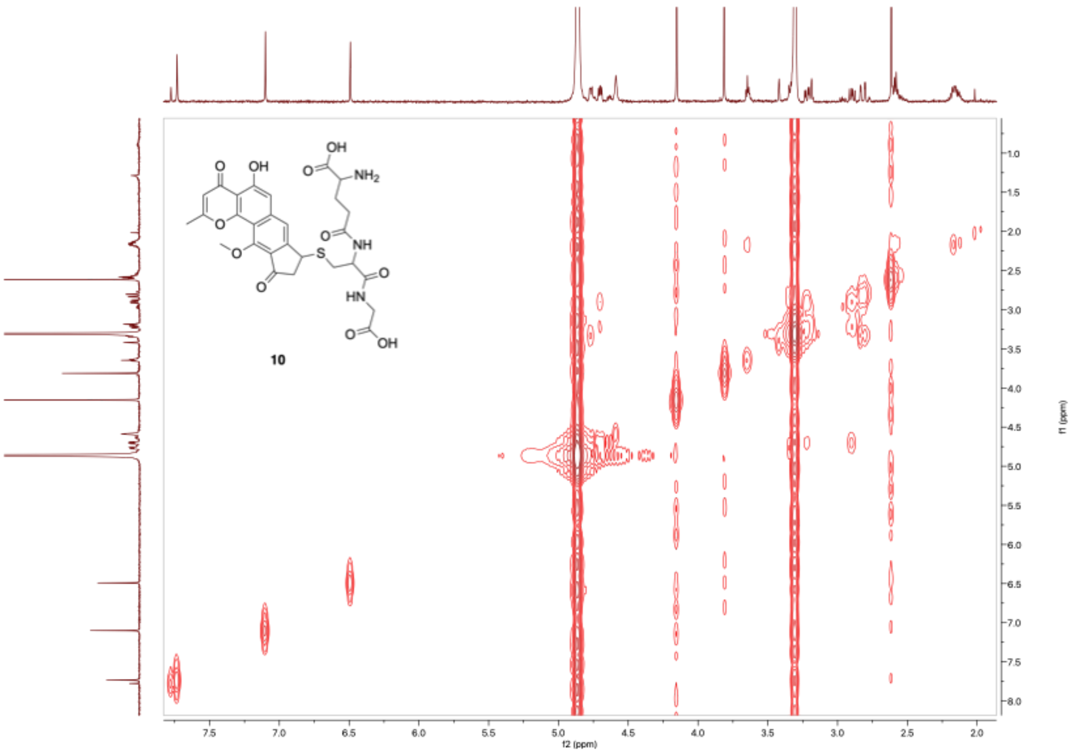

Figure S29: ^{1}H-^{1}H COSY NMR Spectrum of **10** (600MHz, CD_3OD)

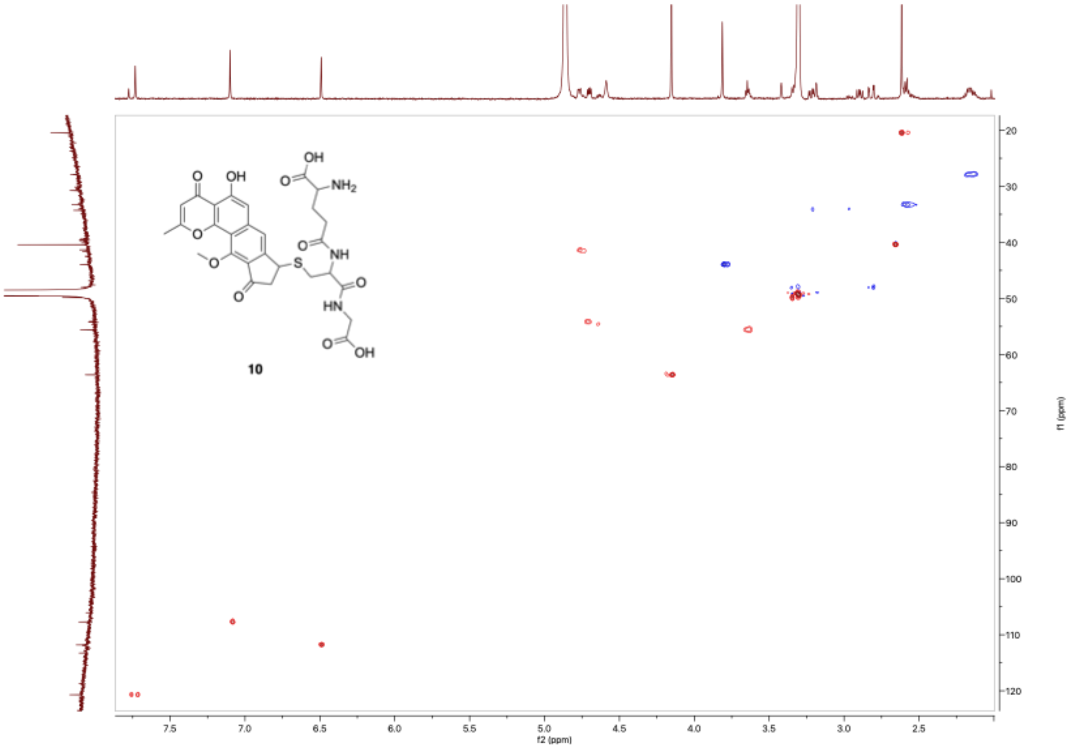

Figure S30: ^{1}H-^{13}C HSQC NMR Spectrum of **10** (600MHz, CD_3OD)

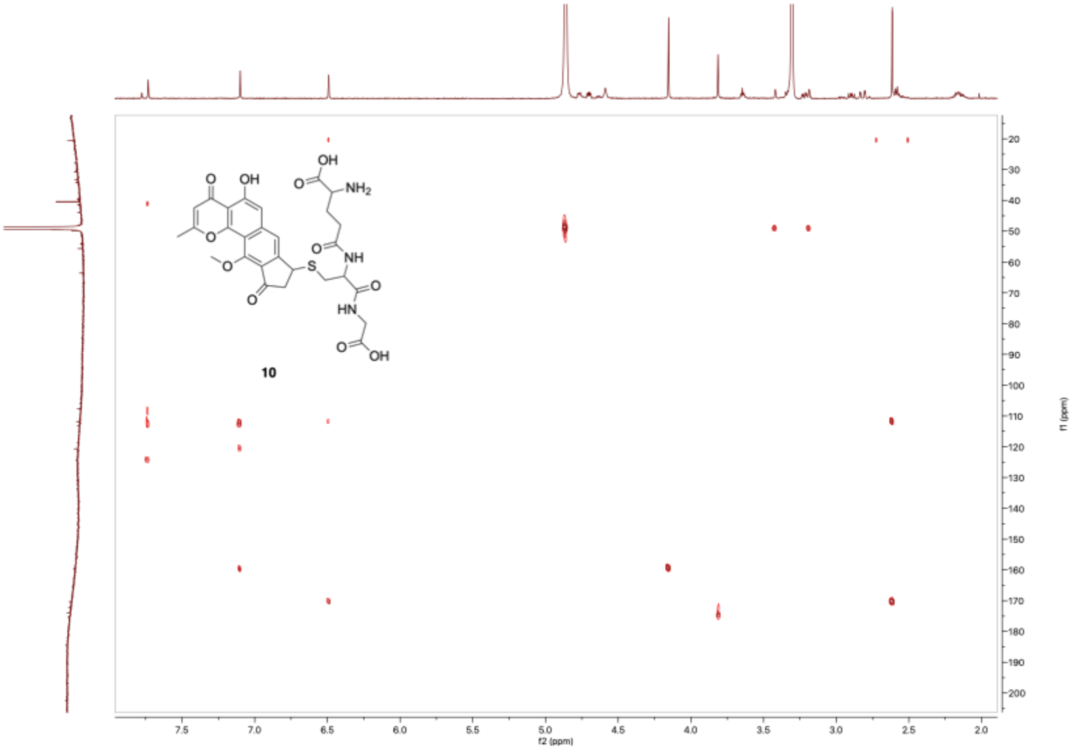

Figure S31: ^{1}H-^{13}C HMBC NMR Spectrum of **10** (600MHz, CD_3OD)

References

(1) Harvey, C. J. B.; Tang, M.; Schlecht, U.; Horecka, J.; Fischer, C. R.; Lin, H.-C.; Li, J.; Naughton, B.; Cherry, J.; Miranda, M.; Li, Y. F.; Chu, A. M.; Hennessy, J. R.; Vandova, G. A.; Inglis, D.; Aiyar, R. S.; Steinmetz, L. M.; Davis, R. W.; Medema, M. H.; Sattely, E.; Khosla, C.; St Onge, R. P.; Tang, Y.; Hillenmeyer, M. E. HEx: A Heterologous Expression Platform for the Discovery of Fungal Natural Products. *Sci Adv* **2018**, *4* (4), eaar5459.

(2) Prjibelski, A.; Antipov, D.; Meleshko, D.; Lapidus, A.; Korobeynikov, A. Using SPAdes De Novo Assembler. *Curr. Protoc. Bioinformatics* **2020**, *70* (1), e102.

(3) Stanke, M.; Diekhans, M.; Baertsch, R.; Haussler, D. Using Native and Syntenically Mapped cDNA Alignments to Improve de Novo Gene Finding. *Bioinformatics* **2008**, *24* (5), 637–644.

(4) Stanke, M.; Schöffmann, O.; Morgenstern, B.; Waack, S. Gene Prediction in Eukaryotes with a Generalized Hidden Markov Model That Uses Hints from External Sources. *BMC Bioinformatics* **2006**, *7*, 62.

(5) Buchfink, B.; Xie, C.; Huson, D. H. Fast and Sensitive Protein Alignment Using DIAMOND. *Nat. Methods* **2015**, *12* (1), 59–60.

(6) Dunbar, K. L.; Perlatti, B.; Liu, N.; Cornelius, A.; Mummau, D.; Chiang, Y.-M.; Hon, L.; Nimavat, M.; Pallas, J.; Kordes, S.; Ng, H. L.; Harvey, C. J. B. Resistance Gene-Guided Genome Mining Reveals the Roseopurpurins as Inhibitors of Cyclin-Dependent Kinases. *Proc. Natl. Acad. Sci. U. S. A.* **2023**, *120* (48), e2310522120.

(7) Helf, M. J.; Fox, B. W.; Artyukhin, A. B.; Zhang, Y. K.; Schroeder, F. C. Comparative Metabolomics with Metaboseek Reveals Functions of a Conserved Fat Metabolism Pathway in C. Elegans. *Nat. Commun.* **2022**, *13* (1), 782.

(8) Huber, F.; van der Burg, S.; van der Hooft, J. J. J.; Ridder, L. MS2DeepScore: A Novel

Deep Learning Similarity Measure to Compare Tandem Mass Spectra. *J. Cheminform.* **2021**, *13* (1), 84.
(9) Xie, Z.; Bailey, A.; Kuleshov, M. V.; Clarke, D. J. B.; Evangelista, J. E.; Jenkins, S. L.; Lachmann, A.; Wojciechowicz, M. L.; Kropiwnicki, E.; Jagodnik, K. M.; Jeon, M.; Ma'ayan, A. Gene Set Knowledge Discovery with Enrichr. *Curr Protoc* **2021**, *1* (3), e90.
(10) Piotrowski, J. S.; Li, S. C.; Deshpande, R.; Simpkins, S. W.; Nelson, J.; Yashiroda, Y.; Barber, J. M.; Safizadeh, H.; Wilson, E.; Okada, H.; Gebre, A. A.; Kubo, K.; Torres, N. P.; LeBlanc, M. A.; Andrusiak, K.; Okamoto, R.; Yoshimura, M.; DeRango-Adem, E.; van Leeuwen, J.; Shirahige, K.; Baryshnikova, A.; Brown, G. W.; Hirano, H.; Costanzo, M.; Andrews, B.; Ohya, Y.; Osada, H.; Yoshida, M.; Myers, C. L.; Boone, C. Errata: Functional Annotation of Chemical Libraries across Diverse Biological Processes. *Nat. Chem. Biol.* **2017**, *13* (12), 1286.
(11) Schmidt, E. K.; Clavarino, G.; Ceppi, M.; Pierre, P. SUnSET, a Nonradioactive Method to Monitor Protein Synthesis. *Nat. Methods* **2009**, *6* (4), 275–277.
(12) Bolger, A. M.; Lohse, M.; Usadel, B. Trimmomatic: A Flexible Trimmer for Illumina Sequence Data. *Bioinformatics* **2014**, *30* (15), 2114–2120.
(13) Dobin, A.; Davis, C. A.; Schlesinger, F.; Drenkow, J.; Zaleski, C.; Jha, S.; Batut, P.; Chaisson, M.; Gingeras, T. R. STAR: Ultrafast Universal RNA-Seq Aligner. *Bioinformatics* **2013**, *29* (1), 15–21.
(14) Anders, S.; Pyl, P. T.; Huber, W. HTSeq--a Python Framework to Work with High-Throughput Sequencing Data. *Bioinformatics* **2015**, *31* (2), 166–169.
(15) Love, M. I.; Huber, W.; Anders, S. Moderated Estimation of Fold Change and Dispersion for RNA-Seq Data with DESeq2. *Genome Biol* **2014**, *15* (12), 550.
(16) Arnone, A.; Camarda, L.; Merlini, L.; Nasini, G. The Structure of Ligustrone A, B and C, New Metabolites of Cercospora Ligustrina Boerema. *Gazz. Chim. Ital.* **1975**, *105* (9), 1093.
(17) Ernst-Russell, M. A.; Chai, C. L.; Wardlaw, J. H.; Elix, J. A. Euplectin and Coneuplectin, New Naphthopyrones from the Lichen Flavoparmelia Euplecta. *J. Nat. Prod.* **2000**, *63* (1), 129–131.
(18) Ishiyama, D.; Kanai, Y.; Senda, H.; Iwatani, W.; Takahashi, H.; Konno, H.; Kanazawa, S. Novel Human Topoisomerase I Inhibitors, Topopyrones A, B, C and D II. Structure Elucidation. *J. Antibiot.* **2000**, *53* (9), 873–878.
